# Characterization, antibacterial, antioxidant, antidiabetic, and anti-inflammatory activities of green synthesized silver nanoparticles using *Phragmanthera austroarabica* A. G. Mill and J. A. Nyberg extract

**DOI:** 10.3389/fmicb.2022.1078061

**Published:** 2023-01-05

**Authors:** Dina M. Khodeer, Ali M. Nasr, Shady A. Swidan, Sarah Shabayek, Roaa M. Khinkar, Mohammed M. Aldurdunji, Maryam A. Ramadan, Jihan M. Badr

**Affiliations:** ^1^Department of Pharmacology and Toxicology, Faculty of Pharmacy, Suez Canal University, Ismailia, Egypt; ^2^Department of Pharmaceutics, Faculty of Pharmacy, Port Said University, Port Said, Egypt; ^3^Department of Pharmaceutics and Industrial Pharmacy, Faculty of Pharmacy, Galala University, Suez, Egypt; ^4^Department of Pharmaceutics and Pharmaceutical Technology, Faculty of Pharmacy, The British University in Egypt, Cairo, Egypt; ^5^The Centre for Drug Research and Development (CDRD), Faculty of Pharmacy, The British University in Egypt, Cairo, Egypt; ^6^Department of Microbiology and Immunology, Faculty of Pharmacy, Suez Canal University, Ismailia, Egypt; ^7^Department of Pharmacy Practice, Faculty of Pharmacy, King Abdulaziz University, Jeddah, Saudi Arabia; ^8^Department of Clinical Pharmacy, College of Pharmacy, Umm Al-Qura University, Makkah, Saudi Arabia; ^9^Department of Pathology, Medical Research Institute, Alexandria University, Alexandria, Egypt; ^10^Department of Pharmacognosy, Faculty of Pharmacy, Suez Canal University, Ismailia, Egypt

**Keywords:** sustainability of natural resources, *Phragmanthera austroarabica*, silver nanoparticle, antioxidant, antidiabetic, antimicrobial activity

## Abstract

**Introduction:**

Diabetes mellitus is a chronic metabolic disorder that exhibited great expansion all over the world. It is becoming an epidemic disease adding a major burden to the health care system, particularly in developing countries.

**Methods:**

The plant under investigation in the current study *Phragmanthera austroarabica* A. G. Mill and J. A. Nyberg is traditionally used in Saudi Arabia for the treatment of diabetes mellitus. The methanolic extract (200 mg/kg) of the plant and pure gallic acid (40 mg/kg), a major metabolite of the plant, as well as their silver nanoparticle formulae (AgNPs) were evaluated for their antidiabetic activity.

**Results and Discussion:**

The results showed a decrease in body fat, obesity, an improvement in lipid profiles, normalization of hyperglycemia, insulin resistance, and hyperinsulinemia, and an improvement in liver tissue structure and function. However, the results obtained from AgNPs for both extract and the pure gallic acid were better in most measured parameters. Additionally, the activity of both the crude extract of the plant and its AgNPs were evaluated against a number of gram-positive, gram-negative bacteria and fungi. Although the activity of the crude extract ranged from moderate to weak or even non-active, the AgNPs of the plant extract clearly enhanced the antimicrobial activity. AgNPs of the extract demonstrated remarkable activity, especially against the Gram-negative pathogens *Proteus vulgaris* (MIC 2.5 μg/ml) and *Pseudomonas aeruginosa* (MIC 5 μg/ml). Furthermore, a promising antimicrobial activity was shown against the Gram-positive pathogen *Streptococcus mutants* (MIC 1.25 μg/ml).

## 1. Introduction

Family Loranthaceae comprises many genera that are characterized by the accumulation of phenolic compounds as major active constituents (Kim et al., 2004; Badr et al., 2013). Additionally, these plants demonstrated solid evidence of antidiabetic, anticancer and antilipidemic activities (Kim et al., 2004; Osadebe et al., 2010; Peter and Obi, 2010). Diabetes triggers the production of reactive oxygen species (ROS) (Savu et al., 2012; Son, 2012). Consequently, agents exhibiting radical-scavenging activity can abolish ROS-induced oxidative damage (Pietta, 2000; Kucharska et al., 2004). *Phragmanthera austroarabica* A. G. Mill and J. A. Nyberg is a semi-parasitic plant belonging to the family Loranthaceae and traditionally used in Saudi Arabia to treat diabetes mellitus (Hanafy and Badr, 2014). Our previous research concerning this plant afforded the isolation and identification of many metabolites with promising biological activity. Additionally, the plant revealed antidiabetic activity that agrees with its traditional use (Bamane et al., 2012; Badr, 2014; Hanafy and Badr, 2014; Aldawsari et al., 2017). Among the significant constituents of *P. austroarabica* is gallic acid, a phenolic compound with potent antioxidant and antidiabetic activities (Bak et al., 2013; Daglia et al., 2014; Abdel-Moneim et al., 2018). Gallic acid is reported to have hypoglycemic activity by activating pancreatic cells (Fatima et al., 2017). Others reported the antioxidant potential of gallic acid as an agent for protection against diabetes-induced diseases (Patel and Goyal, 2011). Variya et al. (2020) suggested its action is due to improved Glut4 translocation leading to improved glucose uptake in 3 T3-L1 cells and stimulation of adipogenesis and lipid accumulation in 3 T3-L1 adipocytes. Additionally, previous investigation of *P. austroarabica* revealed preliminary screening of the antimicrobial activity of the plant extract toward a number of microorganisms. The results exhibited a promising activity against *Staphylococcus aureus* and *Bacillus subtilis* (Waly et al., 2012). Nanotechnology provides a platform to enhance the action of medicinal plant extracts and plant-derived active ingredients, modify their release, and prevent their undesired side effects. Among the most commonly used nano-forms for delivering phytomedicines are silver nanoparticles (AgNPs). Silver curative and protective properties have been known for centuries (Raymond et al., 2008), and recently, AgNPs have proved antimicrobial (Muhammad et al., 2022), anticancer and antidiabetic effects (Muhammad et al., 2022). It was suggested that the antidiabetic activity of AgNPs is related to the efficient inhibitory action of carbohydrate digestive enzymes such as α-amylase and α-glucosidase (Kannan et al., 2016). AgNPs can be synthesized by different methods, either physical, chemical or biological, which is known as biosynthesis. Biosynthesis is also known as green synthesis, which employs unicellular and extracellular biological organisms such as bacteria, algae and plants to act as reducing and capping agents for the synthesized AgNPs. This technique is now widely used as it is relatively simple, cost-effective, scalable and eco-friendly (Oloruntoyin et al., 2022). For these mentioned reasons, our current study aims to apply biosynthesized nanotechnology-based formula to boost both antimicrobial and antidiabetic activities of *P. austroarabica* extract. Different biological parameters are compared with the conventional methanolic plant extract.

## 2. Results and discussion

### 2.1. Quantitative estimation of gallic acid

**Figure 1:**
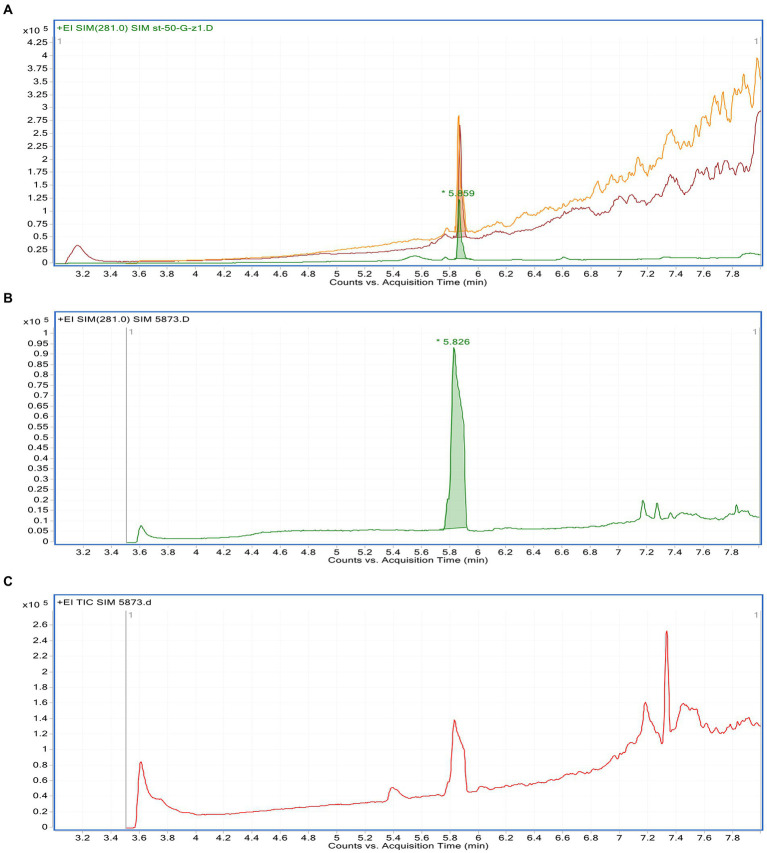
GC–MS chromatograms of: a mixture of methanolic *Phragmanthera austroarabica* crude extract and gallic acid standard solution **(A)**, gallic acid standard solution **(B)**, methanolic *P. austroarabica* crude extract **(C)**.

The linearity of the suggested GC–MS method was inspected by analyzing the standard solution of gallic acid in a series of different concentrations. The calibration curve of gallic acid was obtained by plotting different concentrations against the corresponding peak area (applied in triplicate). Statistical investigation of the received data was verified using linear regression analysis. The linear relationship was obtained over the concentration ranges of 1.5–10.4 μg/ml with a correlation coefficient (*R*^2^) equal 0.9857, and the linear regression equation was *Y* = 3506.3 *X* + 101,700. The limit of detection of gallic acid based on the signal-to-noise approach was 0.18 μg/ml, while the limit of quantitation was 0.41 μg/ml. The precision was determined based on the analysis of three different concentrations of gallic acid applied in triplicate. The method precision was indicated by the low value of RSD (3.1%). The specificity of the method was confirmed as no endogenous interference was detected at the retention times of the analyte (Figure 1). Upon application of the GC–MS method, gallic acid was determined as 44.8 mg/g of *Phragmanthera austroarabica* crude extract.

### 2.2. Total phenolic and antioxidant activity of *Phragmanthera austroarabica*

The phenolic compounds generally play a significant role in the studied biological activities (Tatipamula and Kukavica, 2021). In the current study, total phenolic content of the plant extract was determined as 423.19 ± 19.75 mg/g of dry plant extract. To assess the antioxidant potential of the plant extract, three different methods were implemented. The results demonstrated that *P. austroarabica* crude extract exhibited scavenging activity on DPPH with IC_50_ values of 17.62 ± 3.41 μg/ml while the positive control H_2_O_2_ demonstrated IC_50 =_ 11.58 ± 2.13 μg/ml. Results of FRAP showed that *P. austroarabica* crude extract had promising reduction ability with 1.95 ± 3.41 mMol Fe^+2^/g compared to ascorbic acid as a positive control which showed 2.95 ± 0.91 mMol Fe^+2^/g. Finally, the total antioxidant capacity of the extract and ascorbic acid as a positive control exhibited antioxidant potential equal 31.75 ± 1.23 and 71.28 ± 4.34, respectively. From these data, *P. austroarabica* crude extract could be a promising antioxidant agent.

### 2.3. Biosynthesis of AgNPs using *Phragmanthera austroarabica* crude extract

AgNPs were successfully prepared using the crude extract of the *P. austroarabica*. The plant accumulates a number of important chemical constituents (Badr, 2014). Different secondary metabolites previously isolated from the extract are used as reducing and capping agents. It is well known that polyphenol-rich plant extracts are used in the green synthesis of AgNPs (Oliver et al., 2018; Khedr et al., 2022). Among the different constituents present in *P. austroarabica* extract that plays an important role as reducing and capping agents is catechin. Catechin is a polyphenol that was previously used in the green synthesis of AgNPs (Oliver et al., 2018). Another important constituent in the used extract is gallic acid. Gallic acid is a phenolic acid that is naturally occurring in many plants, fruits, and vegetables and known for its antioxidant activity. Gallic acid was used by Nemčeková and colleagues in the synthesis of AgNPs (Nemčeková et al., 2022). Quercetin also was previously isolated compound from *P. austroarabica* and was involved in the green quercetin-mediated synthesis of AgNPs (Tasca and Riccarda, 2020). Abdallah et al., who synthesized AgNPs using *L. lalambensis* extracts mentioned that some secondary metabolites could be responsible for the reduction and capping of AgNPs. Among these metabolites lupeol and β-sitosterol glucoside, both were isolated from the *P. austroarabica* plant extract used in the current study for reduction and capping of AgNPs (Abdallah and Ali, 2021).

### 2.4. UV-vis absorbance spectroscopy

**Figure 2:**
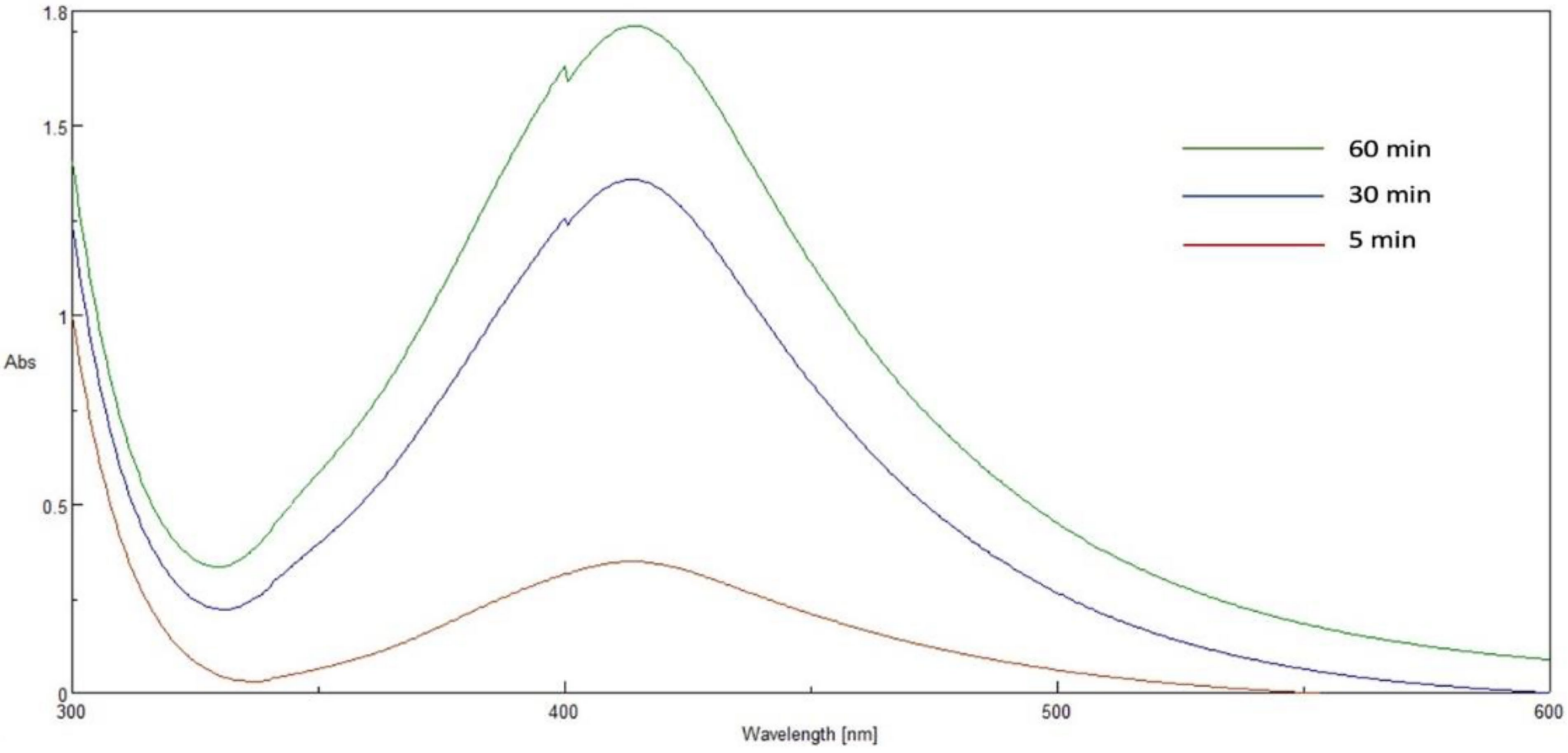
UV–Vis spectrum of *Phragmanthera austroarabica* extract AgNP at different times.

In the current study, the AgNPs were synthesized using *P. austroarabica* crude extract and gallic acid. The extract and gallic acid are used as reducing and capping agents to convert silver ions to atomic form and capping the prepared nanoparticles to enhance the stability of colloids. UV-Vis spectroscopy is a reliable technique to confirm AgNPs formation and monitor nanoparticle dispersion stability. Gallic acid AgNPs dispersion was brown and showed UV-Vis spectrum similar to that obtained by Li et al. (2015). The dispersion was dark yellowish brown for *P. austroarabica* crude extract AgNPs. The color intensity increased over time, as shown in Figure 2. The characteristic surface-plasmon resonance absorption band appeared at 400–500 nm, and the maximum absorbance was at 421 nm. This was similar to several studies such as the carob leaf extract AgNPs prepared by Awwad and colleagues, who found the maximum absorbance at 420 nm (Awwad et al., 2013) and Tripathi et al. (2013), who detected the same wavelength as the current study for AgNPs biosynthesized using Ficus bands leaf extract.

### 2.5. Transmission electron microscopy

**Figure 3:**
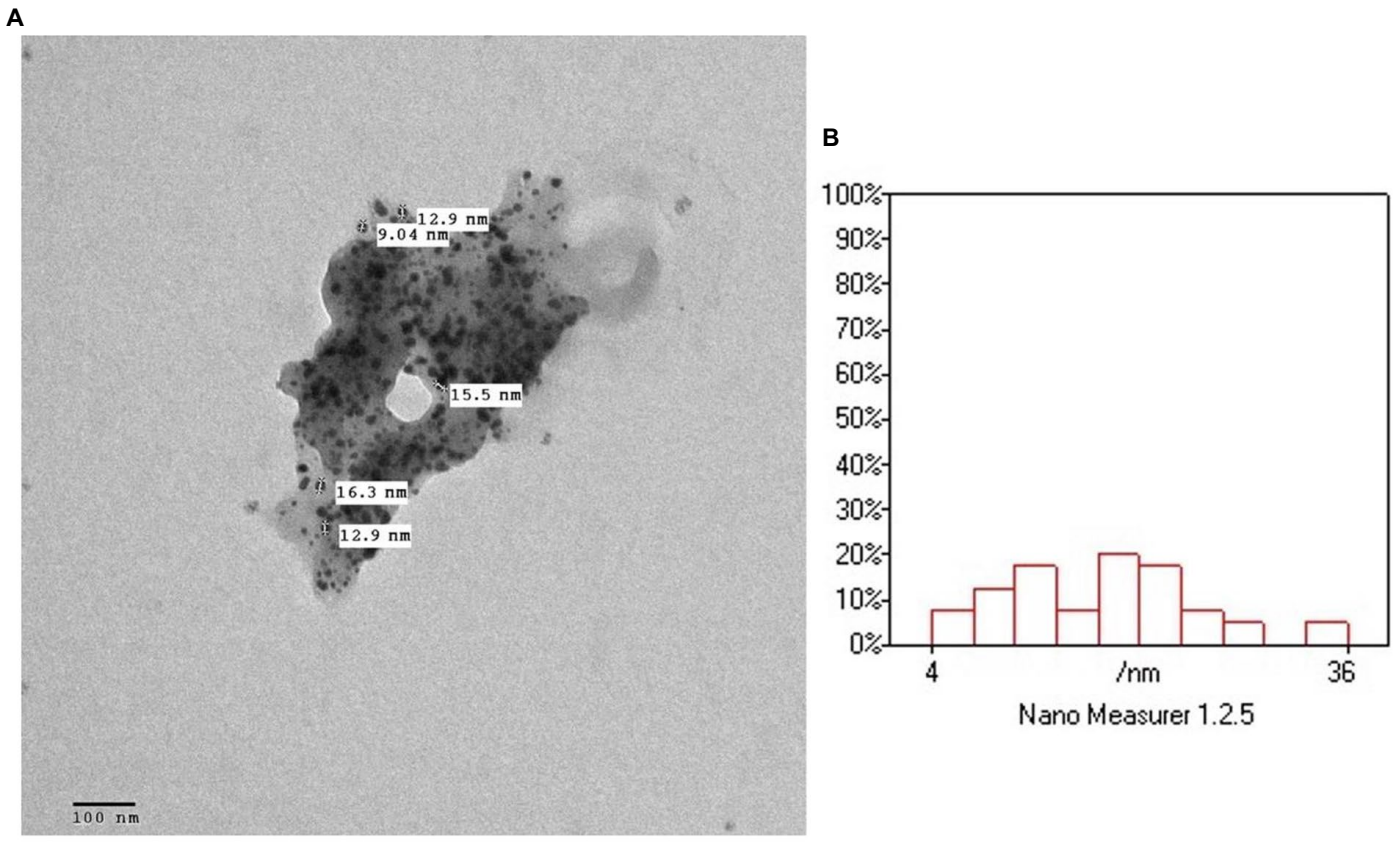
**(A)** TEM micrograph of *Phragmanthera austroarabica* crude extract AgNPs. **(B)** TEM analysis using Nano Measurer software.

The surface morphology, size and shape of the synthesized *P. austroarabica* crude extract AgNPs are investigated by TEM. Figure 3 shows that the AgNPs are spherical, with a minimum particle size of around 13 nm. The TEM technique ensures the dry diameter of the dried sample of the nanoparticles, not the hydrodynamic diameter in the dispersion. High density of the particles appears in the field as an indication of the successful preparation of a large number of AgNPs. Similar size range obtained by Ameen et al. (2020), who prepared AgNPs with green synthesis with cyanobacterium Spirulina platensis, and Suriyakalaa et al. (2013), who prepared biosynthesized AgNPs using Andrographis paniculata, where the TEM revealed the size range from 13 to 27 nm.

### 2.6. AgNPs size and surface charge determination

The DLS is a useful technique for determining the prepared nanoparticles’ hydrodynamic diameter and the surface charge of the particles in the dispersion. Smaller nanoparticles have a higher surface area to volume ratio, enhancing activity. The *P. austroarabica* crude extract AgNPs showed a mean hydrodynamic diameter of 46.22 nm, while gallic acid AgNPs showed a mean diameter of 44.90 nm. Although both preparations were comparable in size, it was noticed that there is a difference in the diameter measured by TEM and DLS techniques. It is clear that they are different techniques, as the former measures the dry diameter in a dried sample, while the latter measures the hydrodynamic diameter of the nanoparticles in dispersion (Cumberland and Jamie, 2009). This finding was also discussed by Diegoli and colleagues who stated that the difference in size between the two techniques is because the DLS technique is sensitive to the double layer surrounding the nanoparticles in dispersion, which is expected to lead to overestimating the mean particle diameter (Diegoli et al., 2008). Khedr et al. (2022), found similar difference in the mean particle size of AgNPs synthesized using *Cynara scolymus* L. crude extracts between the two techniques. Similar results were obtained in the literature where Ahmadi and colleagues prepared AgNPs of *Aloe vera* leaf extract, and the mean diameter was 46 nm (Omid et al., 2018). For gallic acid AgNPs, the diameter value in the current study was intermediate between the value obtained by Li et al. (2015) and by Ahani and Khatibzadeh (2022), which were 17.6 nm, 78.7 nm, respectively. Both studies prepared the nanoparticles at the same pH as the current study (pH 11.0). Regarding the surface charge, a high zeta potential value ensures the stability of colloidal dispersion, which helps the nanocarrier to resist agglomeration leading to sedimentation. When zeta potential is very low, attractive forces overcome repulsion resulting in instability of the colloidal dispersion. So, nanoparticles with higher zeta potential are electrically stabilized (Parmar et al., 2011). The zeta potential of the *P. austroarabica* crude extract AgNPs was-27.4 mV, and the value for gallic acid AgNPs was much higher and was-50.1 mV. These values indicate high stability of the prepared dispersions, especially for the gallic acid AgNPs, which exceeds the value of good stability of-30 mV. Park et al. (2016) who designed green synthesized AgNPs of gallic acid, stated that the zeta potential was −53.47 mV which indicates excellent stability of the system. The zeta potential value of the *P. austroarabica* crude extract AgNPs was comparable to AgNPs prepared by Ezealisiji and coworkers. They biosynthesized the dispersion root bark aqueous extract of Annona muricata, where the zeta potential value was −27.90 mV (Ezealisiji et al., 2017).

### 2.7. Impact on the obesity index and the percentage of weight gain

Compared to the normal group, the diabetic group’s treatment with a high-fat diet and STZ (30 mg/kg) led to a significantly higher final body weight, the percent increase in body weight, and an obesity index at *p* ≤ 0.05 (Table 1). In comparison with the diabetic group, both extract (200 mg/kg) and its corresponding AgNPs for 4 weeks after induction of diabetes significantly ameliorated the % rise in obesity index and body weight (Table 1). In comparison with the diabetic group, gallic acid (40 mg/kg) treated diabetic rats significantly reduced their obesity index %. Conversely, the gallic acid AgNPs formula (40 mg/kg) significantly ameliorated the % increase in obesity index and body weight at *p* ≤ 0.05 (Table 1). Overall, the results achieved by the treatment with the AgNPs formulation for both the extract and the gallic acid were the best in improving the reduction in % change in body weight, final body weight and obesity index (Table 1).

**Table 1:** Effect of *Phragmanthera austroarabica* extract and pure gallic acid versus their AgNPs formulae on the percent increase in obesity parameters.

Group	Final body weight (g)	Obesity index	% Change in body weight	Baseline body weight (g)
Normal	209.5 ± 9.9	0.82 ± 0.06	42.4 ± 9.1	146.9 ± 2.5
Diabetic	353 ± 7.4^a^	5.8 ± 0.5^a^	152 ± 14.3^a^	139.9 ± 5^a^
Diabetic + pioglitazone (10 mg/kg)	226.8 ± 17.7^b^	1.8 ± 0.35^b^	57.9 ± 13.6^b^	143.3 ± 1.4^ab^
Extract (200 mg/kg)	277 ± 17^b^	3.6 ± 0.11^ab^	66 ± 17^b^	167 ± 7^abc^
Extract in AgNPs (200 mg/kg)	173 ± 1.01^bc^	0.86 ± 0.2^bc^	20.1 ± 1.5^b^	144 ± 1^abcd^
Gallic acid (40 mg/kg)	280 ± 6^d^	2.5 ± 0.7^abd^	98.8 ± 43^d^	141 ± 1.01^acde^
Gallic acid in AgNPs (40 mg/kg)	250 ± 5.5^bc^	1.71 ± 0.6^bc^	73.3 ± 8.01^b^	145 ± 10^abcdf^

Obesity prameters are expressed as mean ± S.E and analyzed using one-way ANOVA followed by Bonferroni’s test for multiple comparisons. ^a^*p* ≤ 0.05 versus normal group; ^b^*p* ≤ 0.05 versus Diabetic group; ^c^*p* ≤ 0.05 versus Diabetic + pioglitazone (10 mg/kg); ^d^*p* ≤ 0.05 Diabetic + Extract (200 mg/kg); ^e^*p* ≤ 0.05 versus Diabetic + Extract in AgNPs (200 mg/kg); ^f^*p* ≤ 0.05 versus Diabetic + Gallic acid (40 mg/kg) group, *n* = 5.

### 2.8. Biochemical measurements

According to the current findings, the diabetic group had significantly higher blood glucose levels (Mm/L), serum insulin levels (ng/L), HOMA-IR scores, and serum leptin levels (ng/L) than the normal group at the time of the study *p* ≤ 0.05 (Table 2). However, 4 weeks after diabetes was induced, *P. austroarabica* extract and its AgNPs (200 mg/kg) were administered. This considerably improved the diabetic group’s blood glucose level (Mm/L), serum insulin level (ng/L), HOMA-IR, and serum leptin level (ng/L) at *p* ≤ 0.05 (Table 2). Additionally, when compared to the diabetic group, treatment with gallic acid (40 mg/kg) significantly decreased HOMA-IR, serum insulin, and blood glucose levels Mm/L, Mm/L, and ng/L, respectively (Table 2). Moreover, treatment with the gallic acid (40 mg/kg) in AgNPs significantly decreased blood glucose level (Mm/L), serum insulin level (ng/L), HOMA-IR and serum leptin level (ng/L) in comparison with diabetes. Generally, the results achieved by the treatment with the AgNPs for both the extract and the gallic acid were better than *P. austroarabica* extract and pure gallic acid in improving blood glucose level (Mm/L), serum insulin level (ng/L), HOMA-IR and serum leptin level (ng/L; Table 2).

**Table 2:** Effect of *Phragmanthera austroarabica* extract and pure gallic acid versus their AgNPs formulae on Biochemical Measurements in the experimental groups of type II diabetes in rats.

Group	HOMA-IR	Serum insulin (ng/L)	Blood glucose level (Mm/L)	Serum leptin (ng/L)
Normal	14.4 ± 0.87	2.29 ± 0.06	96 ± 3.8	3.7 ± 0.3
Diabetic	73.99 ± 16.7^a^	7.7 ± 0.28^a^	171 ± 24^a^	13.9 ± 1^a^
Diabetic + pioglitazone (10 mg/kg)	26.4 ± 1.5^b^	3.8 ± 0.02^a,b^	111 ± 5.7 ^b^	4.8 ± 0.3^b^
Extract (200 mg/kg)	21.7 ± 0.73^ab^	4.9 ± 0.06^abc^	101 ± 3.46^b^	11.5 ± 1.01^ab^
Extract in AgNPs (200 mg/kg)	25.13 ± 2.09^ab^	4.5 ± 0.1^abc^	125.3 ± 7.8^b^	5.95 ± 0.05^abc^
Gallic acid (40 mg/kg)	21.9 ± 1.22^ab^	4.43 ± 0.16^abc^	111 ± 2.03^b^	13.25 ± 0.54^acd^
Gallic acid in AgNPs (40 mg/kg)	18.56 ± 0.43^abd^	4.06 ± 0.06^abcd^	102 ± 0.67^b^	7.45 ± 0.15^abce^

Biochemical Measurements are expressed as mean ± S.E.M. and analyzed using one-way ANOVA followed by Bonferroni’s test for multiple comparisons. ^a^*p* ≤ 0.05 versus the normal group; ^b^*p* ≤ 0.05 versus the Diabetic group; ^c^*p* ≤ 0.05 versus Diabetic + pioglitazone (10 mg/kg); ^d^*p* ≤ 0.05 Diabetic + Extract (200 mg/kg); ^e^*p* ≤ 0.05 versus Diabetic + Extract in AgNPs (200 mg/kg); ^f^*p* ≤ 0.05 versus Diabetic + Gallic acid (40 mg/kg) group, *n* = 5.

### 2.9. Liver enzymes

Both the liver index and the serum levels of the liver enzymes AST and ALT were significantly higher in the diabetes group than in the normal group in the current investigation at *p* ≤ 0.05 (Table 3). Additionally, 4 weeks of administration of *P. austroarabica* extract (200 mg/kg) to diabetic rats were able to considerably normalize the liver index as compared to the diabetic group at *p* ≤ 0.05 (Table 3). Additionally, treatment with the *P. austroarabica* AgNPs (200 mg/kg) compared to the diabetes group considerably.

**Table 3:** Effect of *P. austroarabica* extract and pure gallic acid versus their AgNPs on *liver* Enzymes.

Group	Liver index	AST(U/L)	ALT(U/L)
Normal	2.4 ± 0.1	38.9 ± 1	33.8 ± 1.5
Diabetic	3.6 ± 0.2^a^	92.4 ± 0.5^a^	77 ± 2.72^a^
Diabetic + pioglitazone (10 mg/kg)	2.48 ± 0.3^b^	51.1 ± 1.5^a,b^	25.8 ± 2.5^b^
Extract (200 mg/kg)	2.72 ± 0.038^b^	88 ± 4.04^a^	70.5 ± 1.5^a^
Extract in nano formula (200 mg/kg)	3.5 ± 0.036^ac^	44 ± 1.01^bc^	31 ± 1.01^b^
Gallic acid (40 mg/kg)	2.8 ± 0.25^b^	118.5 ± 4.5^abcd^	89.5 ± 1.5^abd^
Gallic acid in nano formula (40 mg/kg)	2.9 ± 0.24^b^	56 ± 2.02^abcde^	37.5 ± 1.5^bce^

Liver Enzymes are expressed as mean ± S.E.M. and analyzed using one-way ANOVA followed by Bonferroni’s test for multiple comparisons. ^a^*p* ≤ 0.05 versus the normal group; ^b^*p* ≤ 0.05 versus the Diabetic group; ^c^*p* ≤ 0.05 versus Diabetic + pioglitazone (10 mg/kg); ^d^*p* ≤ 0.05 Diabetic + Extract (200 mg/kg); ^e^*p* ≤ 0.05 versus Diabetic + Extract in AgNPs (200 mg/kg); ^f^*p* ≤ 0.05 versus Diabetic + Gallic acid (40 mg/kg) group, *n* = 5.

reduced the levels of the two blood liver enzymes ALT and AST at *p* ≤ 0.05 (Table 3). On the other hand, treatment with gallic acid and its AgNPs (40 mg/kg) significantly improved the liver index and serum enzymes ALT and AST levels when compared with diabetic control group at *p* ≤ 0.05 (Table 3).

### 2.10. Lipid profile

When compared to the normal group, the diabetic group had significantly higher levels of serum triglycerides (TG), total cholesterol (TC), and low-density lipoprotein (LDL), and significantly lower levels of high-density lipoprotein (HDL) at *p* ≤ 0.05 (Table 4).

**Table 4:** Effect of *Phragmanthera austroarabica* extract and pure gallic acid versus their AgNPs formulae on lipid profile.

Group	Serum TC (mg/dL)	Serum TG (mg/dL)	LDL (mg/dL)	HDL (mg/dL)
Normal	64 ± 2.99	55.1 ± 3.3	23.9 ± 0.3	46 ± 1.1
Diabetic	116.6 ± 3.3^a^	160 ± 16.3^a^	55.4 ± 4.4^a^	20 ± 2.04^a^
Diabetic + pioglitazone (10 mg/kg)	88.6 ± 1.6^ab^	41.9 ± 2.4^b^	29 ± 1.99^b^	40.4 ± 3.4^b^
Extract (200 mg/kg)	95 ± 2.02^ab^	103.5 ± 2.5^ab^	38 ± 1.01^ab^	39 ± 1.01^b^
Extract in AgNPs (200 mg/kg)	51.5 ± 1.5^abc^	61 ± 1.01^bc^	29.5 ± 0.5^bc^	29.5 ± 0.5
Gallic acid (40 mg/kg)	79.5 ± 0.5^abcd^	86 ± 7.07^ab^	42.5 ± 0.5^abd^	21.5 ± 0.5^ac^
Gallic acid in AgNPs (40 mg/kg)	59.5 ± 1.5^abcde^	95.5 ± 4.5^abd^	37.5 ± 4.5^ab^	34 ± 5.05^b^

Lipid profile are expressed as mean ± S.E.M. and analyzed using one-way ANOVA followed by Bonferroni’s test for multiple comparisons. ^a^*p* ≤ 0.05 versus the normal group; ^b^*p* ≤ 0.05 versus the Diabetic group; ^c^*p* ≤ 0.05 versus Diabetic + pioglitazone (10 mg/kg); ^d^*p* ≤ 0.05 Diabetic + Extract (200 mg/kg); ^e^*p* ≤ 0.05 versus Diabetic + Extract in AgNPs (200 mg/kg); ^f^*p* ≤ 0.05 versus Diabetic + Gallic acid (40 mg/kg) group, *n* = 5.

In comparison to the diabetes group, treatment with the extract (200 mg/kg) for 4 weeks led to a considerable drop in serum triglycerides (TG), total cholesterol (TC), and low-density lipoprotein (LDL), as well as a significant increase in high-density lipoprotein (HDL) at *p* ≤ 0.05 (Table 4). Additionally, when compared to the diabetic group, treatment with the extract (200 mg/kg) in AgNPs for 4 weeks resulted in a substantial drop in serum triglycerides (TG), total cholesterol (TC), and low-density lipoprotein (LDL), as well as a non-significant increase in HDL at *p* ≤ 0.05 (Table 4). Additionally, when compared to the diabetic group, treatment with gallic acid (40 mg/kg) regularly significantly improved serum levels of triglycerides (TG), total cholesterol (TC), and low-density lipoprotein (LDL) at *p* ≤ 0.05 (Table 4). Additionally, compared to the diabetic group, the AgNPs of gallic acid (40 mg/kg) results were the best because they demonstrated a significant reduction in serum triglycerides (TG), total cholesterol (TC), and low-density lipoprotein (LDL), as well as an increase in serum high-density lipoprotein (HDL) at *p* ≤ 0.05 (Table 4).

### 2.11. Hepatic histopathological analysis

**Figure 4:**
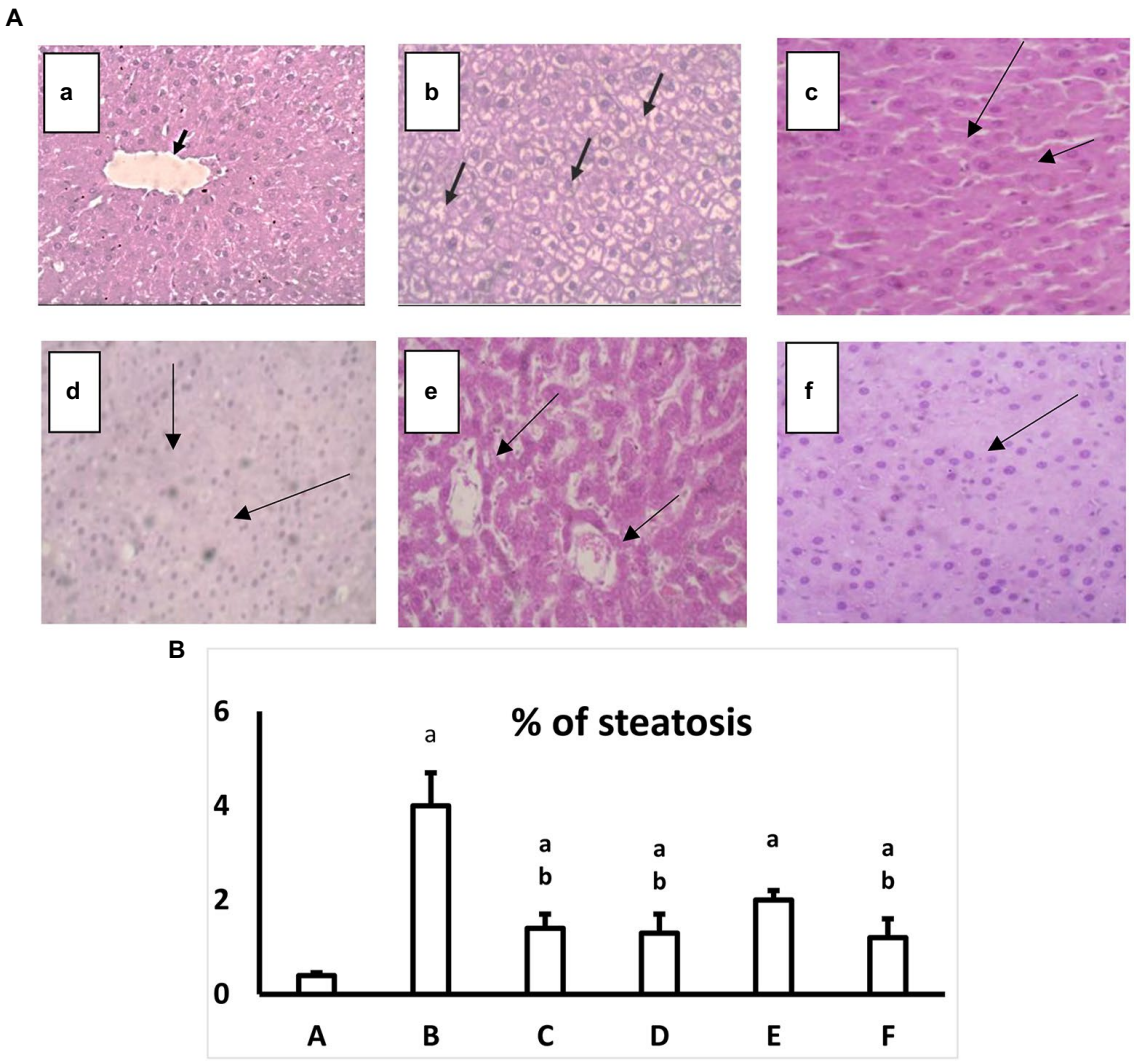
**(A)** Hematoxylin and eosin-stained liver specimen histopathological image at a high magnification ×40. a: Normal group where hepatocytes uniformly distributed in plates radiating from the central vein may be seen in the histopathology photos for liver slices from the normal group (black arrows; H&E, 40×). b: Diabetic group, where hepatocytes exhibit signs of damage, including hydropic degeneration and steatosis (black arrows; H&E, 40×). c: Diabetic+ extract (200 mg/kg) shows Significant hydroponic degeneration in hepatocytes (arrows; H&E, 40×). d: Diabetic+extract in AgNPs (200 mg/kg) group, which shows There is a mild reduction in hydropic degeneration of hepatocytes (Black arrows) with Congested sinusoids (H&E, 40×). e: Diabetic+ Gallic acid (40 mg/kg) the group which shows mild hydropic degeneration (Black arrows), with some hepatocytes, regain uniform morphology (H&E, 40). f: Diabetic+ Gallic acid (40 mg/kg) in AgNPs group shows Uniform hepatocytes (H&E, 40×). **(B)** Effect of regular and AgNPs of the extract versus the pure gallic acid in regular and AgNPs on a percent of liver steatosis. a: Normal group. b: Diabetic group. c: Diabetic+Extract (200 mg/kg). d: Diabetic+Extract (200 mg/kg) in AgNPs. e: Diabetic+ Gallic acid (40 mg/kg). f: Diabetic+ Gallic acid (40 mg/kg) in AgNPs. One-way ANOVA is used to evaluate the data, which is then followed by Bonferroni’s test for multiple comparisons. Results are shown as means S.E.M. ^a^*p* ≤ 0.05 versus the normal group. ^b^*p* ≤ 0.05 versus the Diabetic group, *n* = 5.

The normal group in the current study showed typical liver architecture with uniform morphology. On the other hand, the diabetic group showed no abnormal architecture, lobular inflammation or portal tract injury. All treated groups showed enhancement in liver architecture and a significant decrease in the percent of steatosis compared with the diabetic group at *p* ≤ 0.05. The percent of steatosis and the histopathological abnormalities were improved in both AgNPs formulae for the extract and the pure compound gallic acid (Figures 4A,B).

Induction of diabetes mellitus type II, with a high fat diet followed by a small dose of STZ (30 mg/kg) accompanied with metabolic disorders such as elevation in blood glucose and serum insulin level, with a decrease in the sensitivity of cellular receptors to insulin, on the other hand, there were alterations in serum hepatic enzymes, leptin and lipid profile. So obesity and all of these results came in line with preceding studies which showed the biochemical metabolic abnormalities that concurrently occurred with the incidence of diabetes mellitus type II (Khodeer et al., 2016, 2019; Eltamany et al., 2020; Zeinab et al., 2020).

The displayed auspicious activity of *P. austroarabica* extract could be attributed to its accumulation of many biologically active secondary metabolites. For example, it comprises gallic acid, methyl gallate and catechin, which were also isolated from most of the genera of the family Loranthaceae (Badr et al., 2013, 2016; Badr, 2014). The three compounds are well-known for potent antioxidant activity (Colon and Nerin, 2012; Asnaashari et al., 2014; Zanwar et al., 2014; Badhani et al., 2015; Rahman et al., 2016; Variya et al., 2020; Liu et al., 2021). Additionally, gallic acid, considered a principal constituent of the plant (determined as 44.8 mg/g of the crude dry extract in the current study) was previously announced as a hypoglycemic agent (Adefegha et al., 2015; Xu et al., 2021). Catechin also had an antidiabetic effect (Samarghandian et al., 2017; Mrabti et al., 2018). Other plant constituents which occur in a lower percentage are also phenolic. For examples; chrysophanic acid, emodin, chrysophanic acid-8-O-glucoside, emodin-8-O-glucoside, pectolinarigenin, quercetin, dillenetin-3-O-glucoside and catechin-4’-O-gallate (Badr, 2014). Many previously published studies have shown that phenolics exhibit potential therapeutic benefits in alleviating diabetes and obesity complications (Ademiluyi and Oboh, 2013; Ibrahim et al., 2014; Striegel et al., 2015; Rupasinghe et al., 2017).

According to our study, the whole crude plant extract showed promising antioxidant activity. There is an undoubtful correlation between antioxidant and antidiabetic activity. The plants exerting antioxidant impact can conserve β-cells from reactive oxygen species and consequently prevent the occurrence of diabetic disorders (Patel et al., 2012; Sheela et al., 2013; Sekhon-Loodu and Rupasinghe, 2019). However, the results obtained from AgNPs for both extract and the pure compound gallic acid were the best in most measured parameters.

### 2.12. Antimicrobial activity

**Figure 5:**
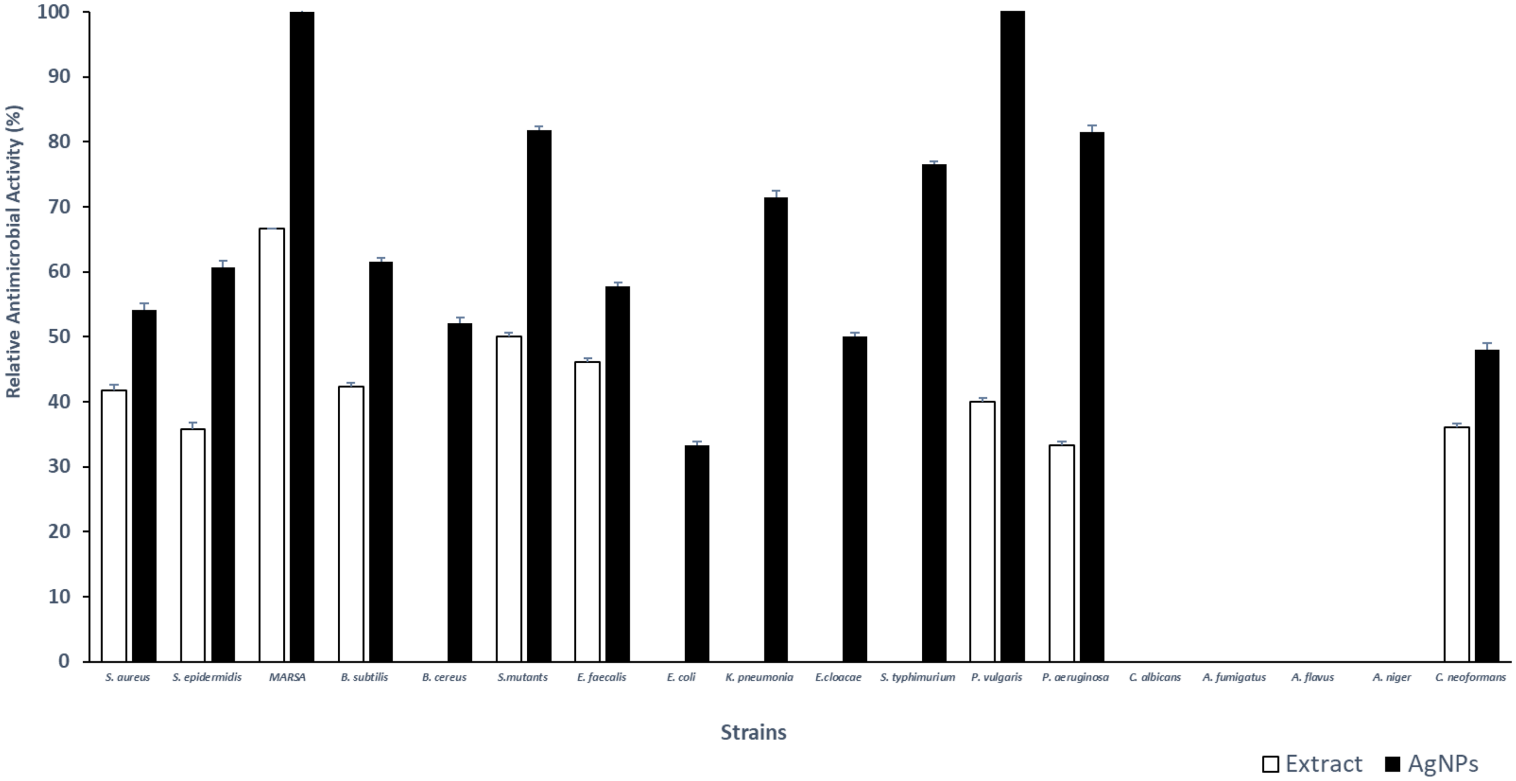
Fold change in Inhibition zone diameters of AgNPs (1 mg/ml) relative to *Phragmanthera austroarabica* extract (10 mg/ml) as obtained using Agar plate diffusion assay.

Total extract of *P. austroarabica* showed weak antimicrobial activity against *S. aureus*, *Staphylococcus epidermidis*, methicillin-resistant *S. aureus* (MRSA), *B. subtilis*, *Streptococcus mutants*, *Enterococcus faecalis*, *Proteus vulgaris*, *Pseudomonas aeruginosa* and, *Cryptococcus neoformans*. The activity of the extract ranged from 33 to 67% relative to the activity of positive control, where the highest antimicrobial activity was shown against MRSA. However, *P. austroarabica* extract showed no antibacterial activity against *Bacillus cereus*, *Escherichia coli*, *Klebsiella pneumoniae*, *Enterobacter cloacae*, *Salmonella typhimurium*. Similarly, *P. austroarabica* extract demonstrated no antifungal activity against *Candida albicans*, *Aspergillus fumigatus*, *Aspergillus flavus*, or *Aspergillus niger*. In consitence with others, there was a remarkable enhancement in the antimicrobial activity of *P. austroarabica* extract (1.3 up to 2.7-fold increase in inhibition zone diameters) when formulated with green synthesized AgNPs using only 10-fold less than the crude extract concentration (Marrez et al., 2019). Inhibition zone diameters of *P. austroarabica* extract and AgNPs obtained using agar plate diffusion assay are shown in Table 5. The fold change in antimicrobial activity of green synthesized AgNPs relative to *P. austroarabica* extract is shown in Figure 5.

**Table 5:** Inhibition zone diameters of *Phragmanthera austroarabica* extract (10 mg/ml) and AgNPs (1 mg/ml) obtained using Agar plate diffusion assay.

Strain	Diameter of inhibition zone (mm)		
	Extract	AgNPs	Positive control
**Gram positive bacteria**			
*Staphylococcus aureus* ATCC 25923	10 ± 1	13 ± 1	24 ± 0
*Staphylococcus epidermidis* RCMB 009-(2)	10 ± 1	17 ± 1	28 ± 1
**Methicillin-Resistant *Staphylococcus aureus* (MRSA) ATCC 4330**	**10** ± 0	**15** ± 1	**15** ± 0.58
*Bacillus subtilis* NRRL B-543	11 ± 0.58	16 ± 0.58	26 ± 1
*Bacillus cereus* RCMB 027-(1)	NA	13 ± 1	25 ± 0
***Streptococcus mutants* ATCC 25175**	**11** ± 0.58	**18** ± 0.58	**22** ± 0.58
*Enterococcus faecalis* ATCC 29212	12 ± 0.58	15 ± 0.58	26 ± 1
**Gram negative bacteria**			
*Escherichia coli* ATCC 25922	NA	10 ± 0.58	30 ± 1
***Klebsiella pneumoniae* ATCC 13883**	**NA**	**15** ± 1	**21** ± 0.58
*Enterobacter cloacae* ATCC 23355	NA	15 ± 0.58	30 ± 0.58
***Salmonella typhimurium* ATCC 14028**	**NA**	**13** ± 0.58	**17** ± 1
***Proteus vulgaris* ATCC 13315**	**10** ±	**27** ± 0.58	**25** ± 1
***Pseudomonas aeruginosa* ATCC 27853**	**9** ±	**22** ± 1	**27** ± 0
**Fungi**			
*Candida albicans* ATCC 10231	NA	NA	20 ± 0.58
*Aspergillus fumigatus* RCMB 002008	NA	NA	17 ± 1
*Aspergillus flavus* RCMB 002002	NA	NA	16 ± 1
*Aspergillus niger* RCMB 002005	NA	NA	15 ± 0
*Cryptococcus neoformans* RCMB 0049001	9 ± 0.58	12 ± 1	25 ± 0

NA, No Antimicrobial Activity. The results shown in bold underwent further testing to determine minimum inhibitory concentration (MIC).

The AgNPs demonstrated remarkable activity against the Gram-negative pathogens *P. vulgaris* (MIC 2.5 μg/ml), *P. aeruginosa* (MIC 5 μg/ml) in addition to *K. pneumoniae* (MIC 10 μg/ml). Furthermore, a promising antimicrobial activity was shown against the Gram-positive pathogen *S. mutants* (MIC 1.25 μg/ml) and MRSA (MIC 10 μg/ml). Minimum inhibitory concentrations (μg/ml) of *P. austroarabica* extract and AgNPs obtained using agar plate diffusion assay are shown in Table 6. Our results coincide with previous reports (Xu and Lee, 2001; Cushnie and Lamb, 2005; Li et al., 2015; Jaisinghani, 2017; Abhishek et al., 2019; Marrez et al., 2019; Yang et al., 2020; Albutti et al., 2021; Nguyen and Bhattacharya, 2022; Qanash et al., 2022) that demonstrated the strong antimicrobial activity of either phenolics (namely gallic acid), flavonoids (in particular quercetin) or plant extract rich in these chemical constituents toward a number of microorganisms including *P. vulgaris*, *P. aeruginosa*, *K. pneumoniae*, and MRSA. These pathogens are well-documented eitological agents that were previously reported to be associated with hospital acquired infections (Khan et al., 2015; Haque et al., 2018; Centers for Disease Control and Prevention (CDC), 2019). The remarkable antimicrobial activitiy of the AgNPs-based extracts suggests its usage as efficective disinfectants in health care yards. Furthermore, similar to others (Yi et al., 2011; Osonga et al., 2019), we demonstrated remakable antimicrobial activity against some oral pathogens such as *S. mutants* and to a lesser extent against *E. faecalis* which play a central role in dental carries and root canal infections (Krzyściak et al., 2014; Blancas et al., 2021; Tu et al., 2022). This implays the potential benefits of the AgNPs-based extracts in preventing dental diseases and promoting oral health. The *P. austroarabica* extract under investigation in the current study is rich in gallic acid and quercetin. Based on previous literature (Simoes et al., 2009; Borges et al., 2013; Kang et al., 2018; Osonga et al., 2019; Alvarado-Martinez et al., 2020), the proposed antibaterial activityl activity of these compounds is primairly through disruption of outer membrabe integrity and permeabilization. Additional antibacterial activity has been reported for quercetin through targetting DNA gyrase and interfering with nucleic acid synthesis (Simoes et al., 2009; Osonga et al., 2019). Moreover, antibiofilm activity aginst oral pathogens forming dental plaques have also been demonstrated (Albutti et al., 2021). Furthermore, anti-proteus activity of natural plant extracts rich in gallic acid and quercetin have also been reported for *Proteus mirabilis*, a principle cause for urinary tract infections and kidney stone formations. Smanthong et al. (2022) have shown the ethanolic extract of *Sida acuta* Burm. F. which is rich in gallic acid and quercetin, to exhibit potent antivirulent activity through interfering with swarming motility and urase activity of *P. mirabilis*. In addition, *Sida acuta* Burm. F. extract showed modest potency on anti-struvite crystallization, hence interfering with kidney stone formation. In the present study, we have shown remarkable antimicrobial activity of the AgNPs-based extract of *P. austroarabica* against *P. vulgaris*. A similar mechanism of action can be hyposized against *P. vulgaris* as both *P. mirabilis* and *P. vulgaris* account for the vast majority of *proteus* species clinical isolates, in particular nosocomial urinary tract infections. They both are urease positive and display swarming motility as major virulence factors (Bennett et al., 2019).

**Table 6:** Minimum inhibitory concentrations (μg/ml) of *Phragmanthera austroarabica* extract (10 mg/ml) and AgNPs (1 mg/ml) obtained using Agar plate diffusion assay.

Strain	Minimum inhibitory concentration (μg/ml)
Methicillin-Resistant *Staphylococcus aureus* (MRSA) ATCC 4330	10
*Streptococcus mutants* ATCC 25175	1.25
*Klebsiella pneumoniae* ATCC 13883	10
*Salmonella typhimurium* ATCC 14028	10
*Proteus vulgaris* ATCC 13315	2.5
*Pseudomonas aeruginosa* ATCC 27853	5

## 3. Materials and methods

### 3.1. Plant material and extraction process

*P. austroarabica* was gathered from Abha, in the Southwestern region of King of Saudi Arabia. The plant was taxonomically identified at the Faculty of Science, King Abdulaziz University. To prepare the extract, 600 *g* of the dry plant were finely powdered and extracted with methyl alcohol (4 × 1,000 ml) at ambient temperature, then concentrated under vacuum by rotary evaporator to give finally 75 *g* of dry extract. The dry extract was maintained in a refrigerator till use.

### 3.2. Preparation of standard solution

The analytical standard (gallic acid) was obtained from Sigma Chemical Co. Stock solution was prepared by accurately weighing 100 mg of gallic acid, dissolved in 10 ml of methanol, then the volume was adjusted to 100 ml. Serial dilutions were prepared using this stock solution to construct the calibration graph.

### 3.3. Determination of gallic acid using GS-MS

Quantitation of gallic acid was achieved by GC (Agilent Technologies 7890A) interfaced with a mass-selective detector (MSD, Agilent 7000) equipped with a polar Agilent DB-5 ms (5%-phenyl methyl poly siloxane) capillary column (30 m × 0.25 mm i. d. and 0.25 μm film thickness). Helium was the carrier gas, and the linear velocity was 1 ml/min. The temperatures of the injector and detector were 200°C and 250°C, respectively. The injected volume was 1 μl. Mass operating parameters were 70 eV for ionization potential and 250°C as interface temperature.

### 3.4. Determination of the antioxidant activity of *Phragmanthera austroarabica*

The antioxidant activity of the plant extract was assessed using the DPPH free radical scavenging test as previously described (Yen and Duh, 1994; Eltamany et al., 2020). Data was recorded at intervals of one min. to measure the decline in absorbance at 515 nm, and H_2_O_2_ was used as a positive control.

The extract performed the Ferric reducing antioxidant power assay (FRAP) spectrophotometrically according to the previously described method (Oyaizu, 1986; Ferreira et al., 2007). Absorbance was assessed at 700 nm, and ascorbic acid was used as a positive control. The data were displayed as mMol Fe+2 equivalent/g dry sample.

The extract’s total antioxidant capacities (TAC) were defined spectrophotometrically using phosphomolybdenum assay. The method was done as previously declared (Prieto et al., 1999; Eltamany et al., 2022). The absorbance was measured at 695 nm, and the effect was expressed as mg equivalents of gallic per g of extract (mg GAE/g). In all the measurements, UV/Vis spectrophotometer (Milton Roy, Spectronic 1,201, Houston, TX, United States) was utilized.

### 3.5. Determination of total phenolic content of *Phragmanthera austroarabica*

The total phenolic contents of *P. austroarabica* extract was assessed by the Folin–Ciocalteu colourimetric method as previously reported (Prieto et al., 1999).

### 3.6. Biosynthesis and preparation of silver nanoparticles

The biosynthesis of AgNPs of the total extract of *P. austroarabica* was done using a modified method, as previously reported by Mortazavi-Derazkola et al. (2021), with slight modifications. Initially, 10 mg of the extract were dissolved in 1 ml ethanol, then added to 10 ml of 10 mM AgNO_3_. The pH of the solution is adjusted by adding drops of 1 M NaOH. The mixture was agitated in dark at 600 rpm for 60 min at room temperature. The color was changed gradually till yellowish brown, and the color change was observed at 5, 30 and 60 min.

For gallic acid coated AgNPs, the method used by Li et al. (2015) was used. Briefly, 4 ml of 10 mM AgNO_3_ were added to 22 ml of double-distilled water under magnetic stirring at room temperature. Then, 4 ml of 10 mM gallic acid were added, and the pH value was adjusted to 11.0 with 1 M NaOH. Subsequently, the reaction was maintained at room temperature for 30 min.

The AgNPs of both *P. austroarabica* extract and gallic acid were purified by the same procedure. Centrifugation at 15,000 rpm for 1 h. at 4°C was done using a cooling centrifuge (PRO-Research K241R; Centurion, West Sussex, United Kingdom). The AgNPs were re-dispersed in double-distilled water and sonicated for 30 s in the sonicating water bath. Then centrifuged at 15,000 rpm for 1 h at 4°C. The washing procedures using double-distilled water were repeated three times, and then the nanoparticles were stored at 4°C for further investigations.

### 3.7. Characterization of silver nanoparticles

#### 3.7.1. UV-vis absorbance spectroscopy

The UV-Vis spectrum of the prepared AgNPs at predetermined time intervals was acquired using a double-beam spectrophotometer (V630, Jasco, Tokyo, Japan). The spectrum was recorded throughout a range of 300–600 nm.

#### 3.7.2. Transmission electron microscopy

The morphology and the size of the prepared AgNPs were examined using transmission electron microscopy (TEM). The samples were further diluted 50 times with double distilled water. Then the diluted samples were negatively stained with phosphotungstic acid and dried on carbon-coated copper grids. The thin film formed was air-dried at room temperature and observed using a transmission electron microscope (JTEM model 1,010, JEOL^®^, Tokyo, Japan) with an accelerating voltage of about 80 kV. The size distribution of AgNPs was analyzed using Nano measurer 1.2.5 software.

#### 3.7.3. Size analysis and surface charge determination

Average particle size expressed as Z-average, particle size distribution, is described by polydispersity index (PDI) and zeta potential, indicating a surface charge. Furthermore, they were measured using the dynamic light scattering technique (DLS) by Malvern Zetasizer (Nano ZS, Malvern Instruments Ltd., Malvern, United Kingdom). Before analysis, samples were diluted 20 times with distilled water and added to the cell for measurement. All measurements were performed at ambient temperature (25°C).

### 3.8. Antidiabetic activity

#### 3.8.1. Animal experimentation

In the current investigation, 35 male Wistar rats were employed. The body’s initial weight was anywhere between 130 and 170 g. They were kept in clean cages at 21.6°C with regular light and dark cycles. They were given unrestricted access to water and a regular diet or HFD. Under 202209RA2, the Suez Canal University Research Ethics Committee accepted the study’s protocol.

#### 3.8.2. Experimental design

Wistar male rats were distributed into six groups, with 5 rats in every group. During the experimentation period, the first group of rats was designated as the normal group and fed a typical chow diet (14 weeks). The other five groups had a diet of HFD (regular diet, lard fat, glucose 7:1:2) for 7 weeks before receiving a low dose of streptozotocin (STZ) of 30 mg/kg (Srinivasan et al., 2005). Rats in all groups had their fasting blood glucose levels measured after 5 days of administration of STZ. Each rat had blood drawn from the tip of its tail, and an automated blood glucometer was used to measure each animal’s fasting blood sugar (Super Glucocard, Japan). Then for a further 4 weeks rats in Group II (diabetic control group) were given distilled water (1 ml/kg/day, p.o.). Rats in group III were given the methanolic extract of *P. austroarabica* (200 mg/kg/day, p.o.), while rats in group IV were given the AgNPs formula of *P. austroarabica* (200 mg/kg/day, p.o.). Groups V and VII got gallic acid extract and AgNPs (40 mg/kg). The final body weight was collected when the treatment regimens were finished. The following equation was used to compute the change in body weight.


$$\begin{array}{l} △\mathrm{Body weight}=[(\mathrm{The}\;\mathrm{final}\;\mathrm{body}\;\mathrm{weight}-\\ \mathrm{the}\;\mathrm{initial}\;\mathrm{body}\;\mathrm{weight)/initialbodyweight}]*100 \end{array}$$

The obesity index was similarly determined by


$$\begin{array}{l} \mathrm{Obesity}\;\mathrm{index = (weight}\;\mathrm{of}\;\mathrm{total}\;\mathrm{adipose}\;\mathrm{tissue}/\\ \;\;\;\;\;\;\;\;\;\;\;\;\;\;\;\;\;\;\;\;\;\;\;\;\;\;\;\;\;\;\;\;\;\;\;\;\;\;\;\mathrm{final}\;\mathrm{body}\;\mathrm{weight}\times 100)\; \end{array}$$

#### 3.8.3. Processing of liver

Rats were sacrificed under anesthesia. Each rat’s liver was removed and washed with a cold saline solution. After weighing the livers, the following formula was used to calculate the liver index: (liver weight/body weight × 100).

The largest hepatic lobe’s portion of liver tissue was extracted, formaldehyde-fixed, then hematoxylin–eosin-stained tissue.

#### 3.8.4. Biochemical measurements

##### 3.8.4.1. Liver enzymes

Alanine transaminase enzyme (ALT) and aspartate transaminase enzyme (AST) are measured in accordance to (Pan et al., 2006).

##### 3.8.4.2. Lipid profile

Serum total cholesterol (TC), triglycerides (TGs), high-density lipoprotein (HDL), and low-density lipoprotein (LDL), according to (Allain et al., 1974; Fossati and Prencipe, 1982).

##### 3.8.4.3. Insulin, leptin and HOMA-IR

Following the manufacturer’s instructions, the rat insulin and leptin kits were used to measure insulin and leptin serum levels. Insulin resistance was measured using HOMA-IR model (Matthews et al., 1985).

### 3.9. Antimicrobial assay

The antimicrobial activity was screened against Gram-positive bacteria (*S. aureus* ATCC 25923, *S. epidermidis* RCMB 009-(2), Methicillin-Resistant *S. aureus* (MRSA) ATCC 4330, *B. subtilis* NRRL B-543, *B. cereus* RCMB 027-(1), *S. mutants* ATCC 25175, *E. faecalis* ATCC 29212), Gram-negative bacteria (*E. coli* ATCC 25922, *K. pneumoniae* ATCC 13883, *E. cloacae* ATCC 23355, *S. typhimurium* ATCC 14028, *P. vulgaris* ATCC 13315, *P. aeruginosa* ATCC 27853) and fungi (*C. albicans* ATCC 10231, *A. fumigatus* RCMB 002008, *A. flavus* RCMB 002002, *A. niger* RCMB 002005, *C. neoformans* RCMB 0049001). These strains were obtained from the Regional Center for Mycology, and Biotechnology (RCMB), Al-Azhar University, Cairo, Egypt. Bacterial strains were maintained in Brain-Heart infusion broth, and fungal strains were maintained in Potato-Dextrose broth. Agar plate diffusion assay was used to screen the antibacterial and antifungal activities as described before (Tomás et al., 2003; Magaldi et al., 2004; Rojas et al., 2006; Valgas et al., 2007; Synytsya et al., 2017; Gonelimali et al., 2018) with some modifications. Briefly, bacterial suspensions with an inoculum size of of 1 × 10^8^ CFU/ml were prepared by making a direct suspension of fresh overnight colonies in phosphate-buffered saline. Bacterial inocula were prepared to a density equivalent to a 0.5 McFarland standard. Fungal inocula were prepared using 48 h cultures of potato dextrose broth and adjusted to a spore density of 10^6^ spores/ml. A sterile cotton swab was dipped into the adjusted suspension. The dried surface of Mueller-Hinton agar plate was inoculated by streaking the swab over the entire sterile agar surface. Holes with diameters of 6 mm were cut into inoculated plates. Holes were then filled with 100 μl aliquots *P. austroarabica* extract (10 mg/ml) and AgNPs formula (1 mg/ml). The plates were incubated at room temperature for 5 h and then incubated at 37°C for 24 h for bacteria and 48 h for fungi. A clear inhibition zone of > 6 mm diameter was defined as a positive result. A concentration of 4 μg/ml of Gentamicin was a positive control for antibacterial activity, and Ketoconazole at a concentration of 100 μg/ml served as a positive control of antifungal activity. DMSO was a negative control for *P. austroarabica* extract, and distilled water was used as a negative control for AgNPs formula. Strains showing positive results were further tested using holes filled with two-fold serial dilutions (10, 5, 2.5, 1.25, 0.625, 0.3125, 0.15625, 0.078, 0.039, and 0.019 mg/ml) to determine the minimum inhibitory concentration (MIC). Only strains showing inhibition zone diameters comparable to the positive controls (70% or more relative to positive controls) underwent further MIC testing. MIC was recorded as the lowest concentration inhibiting bacterial growth.

### 3.10. Statistical analysis of the data

Results from the current study were analyzed using the SPSS program version 16 and presented as mean ± S.E.M. Quantitative variables were analyzed using a one-way analysis of variance (ANOVA), which was then followed by the Bonferroni multiple comparison test. Significant differences were determined at *p* ≤ 0.05.

## 4. Conclusion

The AgNPs prepared using the *P. austroarabica* extract and the phenolic compound gallic acid as reducing and capping agents are small in size, uniform in shape and of uniform size distribution. The nanoparticles show a high negative charge on the surface, indicating high colloidal particles’ stability. The presence of the extract or gallic acid on the surface of nanoparticles offers a high surface-to-volume ratio, enhancing their pharmacological activity. Our study deduced that the application of AgNPs was a promising technique for enhancing the efficiency of *P. austroarabica* extract and gallic acid as antidiabetic agents. These formulae ameliorated body weight, lipid profile, insulin resistance, hyperinsulinemia, liver tissue structure and function. Moreover, the AgNPs formula of the crude extract of *P. austroarabica* potentiated the activity against the Gram-negative pathogens *P. vulgaris*, *P. aeruginosa*, and the Gram-positive pathogen *S. mutants*.

## Data availability statement

The original contributions presented in the study are included in the article/supplementary material, further inquiries can be directed to the corresponding authors.

## Ethics statement

The study protocol was approved by the ethical committee of the Faculty of Pharmacy at Suez Canal University (approval number: 202109RA2).

## Author contributions

JB, DK, and SaS: conceptualization. DK, AN, ShS, and SaS: methodology. JB, DK, AN, ShS, MR, and SaS: validation. DK, AN, ShS, RK, MR, and MA: formal analysis. RK and MA: resources. JB and DK: data curation. DK, AN, ShS, and SaS: writing—original draft preparation. JB, DK, and SaS: writing—review and editing. JB, AN, and ShS: supervision. JB, DK, and AN: project administration. RK and MA: funding acquisition. All authors have read and agreed to the published version of the manuscript.

## Funding

This research was funded by the Deanship of Scientific Research (DSR) at King Abdulaziz University (KAU), Jeddah, Saudi Arabia, under grant number (RG-29-166-43).

## Conflict of interest

The authors declare that the research was conducted in the absence of any commercial or financial relationships that could be construed as a potential conflict of interest.

## Publisher’s note

All claims expressed in this article are solely those of the authors and do not necessarily represent those of their affiliated organizations, or those of the publisher, the editors and the reviewers. Any product that may be evaluated in this article, or claim that may be made by its manufacturer, is not guaranteed or endorsed by the publisher.

## References

[ref1] Abdallah B. M. Ali E. M. (2021). Green synthesis of silver nanoparticles using the *Lotus lalambensis* aqueous leaf extract and their anti-candidal activity against oral candidiasis. ACS Omega 6, 8151–8162. doi: 10.1021/acsomega.0c06009, PMID: 33817474 PMC8014928

[ref2] Abdel-Moneim A. AbdEl-Twab S. M. Yousef A. I. AbdelReheim E. S. Ashour M. B. (2018). Modulation of hyperglycemia and dyslipidemia in experimental type 2 diabetes by gallic acid and p-coumaric acid: the role of adipocytokines and PPARγ. Biomed. Pharmacother. 105, 1091–1097. doi: 10.1016/j.biopha.2018.06.096, PMID: 30021345

[ref3] Abhishek R. U. Venkatesh H. N. Sudharshana T. N. Mohana D. C. (2019). Antimicrobial activity of 4-O-methylgallic acid isolated from *Phyllanthus polyphyllus* L. against foodborne pathogenic bacteria and yeasts. J. Biological. Act. Prod. Nature 9, 120–134. doi: 10.1080/22311866.2019.1605932

[ref4] Adefegha S. A. Oboh G. Ejakpovi I. I. Oyeleye S. I. (2015). Antioxidant and antidiabetic effects of gallic and protocatechuic acids: a structure–function perspective. Comp. Clin. Path. 24, 1579–1585. doi: 10.1007/s00580-015-2119-7

[ref5] Ademiluyi A. O. Oboh G. (2013). Soybean phenolic-rich extracts inhibit key-enzymes linked to type 2 diabetes (α-amylase and α-glucosidase) and hypertension (angiotensin I converting enzyme) in vitro. Exp. Toxicol. Pathol. 65, 305–309. doi: 10.1016/j.etp.2011.09.005, PMID: 22005499

[ref6] Ahani M. Khatibzadeh M. (2022). Green synthesis of silver nanoparticles using gallic acid as reducing and capping agent: effect of pH and gallic acid concentration on average particle size and stability. Inorgan. Nano Metal Chem. 52, 234–240. doi: 10.1080/24701556.2021.1891428

[ref7] Albutti A. Gul M. S. Siddiqui M. F. Maqbool F. Adnan F. Ullah I. (2021). Combating biofilm by targeting its formation and dispersal using Gallic acid against single and multispecies bacteria causing dental plaque. Pathogens 10:1486. doi: 10.3390/pathogens10111486 34832641 PMC8618234

[ref8] Aldawsari H. M. Eid B. G. Neamatallah T. Zaitone S. A. Badr J. M. (2017). Anticonvulsant and neuroprotective activities of *Phragmanthera austroarabica* extract in pentylenetetrazole-kindled mice. Evid. Based Complement. Alternat. Med. 2017, 1–12. doi: 10.1155/2017/5148219, PMID: 28465705 PMC5390588

[ref9] Allain C. C. Poon L. S. Chan C. S. Richmond W. Fu P. C. (1974). Enzymatic determination of total serum cholesterol. Clin. Chem. 20, 470–475. doi: 10.1093/clinchem/20.4.470 4818200

[ref10] Alvarado-Martinez Z. Bravo P. Kennedy N.-F. Krishna M. Hussain S. Young A. C. (2020). Antimicrobial and antivirulence impacts of phenolics on *Salmonella enterica* serovar Typhimurium. Antibiotics 9:668. doi: 10.3390/antibiotics9100668 33022945 PMC7600263

[ref11] Ameen F. Abdullah M. M. Al-Homaidan A. A. Al-Lohedan H. A. Al-Ghanayem A. A. Almansob A. (2020). Fabrication of silver nanoparticles employing the cyanobacterium Spirulina platensis and its bactericidal effect against opportunistic nosocomial pathogens of the respiratory tract. J. Mol. Struct. 1217:128392. doi: 10.1016/j.molstruc.2020.128392

[ref12] Asnaashari M. Farhoosh R. Sharif A. (2014). Antioxidant activity of gallic acid and methyl gallate in triacylglycerols of Kilka fish oil and its oil-in-water emulsion. Food Chem. 159, 439–444. doi: 10.1016/j.foodchem.2014.03.038, PMID: 24767079

[ref13] Awwad A. K. Salem N. M. Abdeen A. O. (2013). Green synthesis of silver nanoparticles using carob leaf extract and its antibacterial activity. Int. J. Ind. Chem. 4, 1–6. doi: 10.1186/2228-5547-4-29

[ref14] Badhani B. Sharmaa N. Kakkar R. (2015). Gallic acid: a versatile antioxidant with promising therapeutic and industrial applications. RSC Adv. 5, 27540–27557. doi: 10.1039/C5RA01911G

[ref15] Badr J. M. (2014). Chemical constituents of *Phragmanthera austroarabica* A. G. Mill and J. A. Nyberg with potent antioxidant activity. Pharm. Res. 7, 335–340. doi: 10.4103/0974-8490.158436, PMID: 26692747 PMC4660512

[ref16] Badr J. M. Ibrahim S. R. M. Abou-Hussein D. R. (2016). Plicosepalin a, a new antioxidant catechin–gallic acid derivative of inositol from the mistletoe *Plicosepalus curviflorus*. Z. Naturforsch. 71, 375–380. doi: 10.1515/znc-2015-0231 27206319

[ref17] Badr J. M. Shaala L. A. Youssef D. T. A. (2013). Loranthin: a new polyhydroxylated flavanocoumarin from *Plicosepalus acacia* with significant free radical scavenging and antimicrobial activity. Phytochem. Lett. 6, 113–117. doi: 10.1016/j.phytol.2012.11.008

[ref18] Bak E. J. Kim J. Jang S. Woo G. H. Yoon H. G. Yoo Y. J. (2013). Gallic acid improves glucose tolerance and triglyceride concentration in diet-induced obesity mice. Scand. J. Clin. Lab. Invest. 73, 607–614. doi: 10.3109/00365513.2013.831470, PMID: 24219649

[ref19] Bamane F. H. Badr J. M. Amin O. R. M. (2012). Antioxidant activities and flavonoid contents of selected plants belonging to family Loranthaceae. Afr. J. Biotechnol. 11, 14380–14385. doi: 10.5897/AJB12.2093

[ref20] Bennett J. E. Dolin R. Blaser M. J. (2019). Mandell, Douglas, and Bennett's Principles and Practice of Infectious Diseases E-Book. Philadelphia, PA: Elsevier Health Sciences.

[ref21] Blancas B. Lanzagorta M. D. L. Jiménez-Garcia L. F. Lara R. Molinari J. L. Fernandez A. M. J. C. (2021). Study of the ultrastructure of *Enterococcus faecalis* and *Streptococcus mutans* incubated with salivary antimicrobial peptides. Clin. Exp. Dent. Res. 7, 365–375. doi: 10.1002/cre2.430 33951334 PMC8204031

[ref22] Borges A. Ferreira C. Saavedra M. J. Simões M. J. (2013). Antibacterial activity and mode of action of ferulic and gallic acids against pathogenic bacteria. Microb. Drug. Resist. 19, 256–265. doi: 10.1089/mdr.2012.0244 23480526

[ref23] Centers for Disease Control and Prevention (CDC), (2019). Health care associated infections (HAIs). Available at: https://www.cdc.gov/hai/organisms/organisms.html (Accessed November 26, 2022).

[ref24] Colon M. Nerin C. (2012). Role of catechins in the antioxidant capacity of an active film containing green tea, green coffee, and grapefruit extracts. Agric. Food Chem. 60, 9842–9849. doi: 10.1021/jf302477y, PMID: 22973940

[ref25] Cumberland S. A. Jamie R. L. (2009). Particle size distributions of silver nanoparticles at environmentally relevant conditions. J. Chromatogr. A 1216, 9099–9105. doi: 10.1016/j.chroma.2009.07.021, PMID: 19647834

[ref26] Cushnie T. P. T. Lamb A. J. (2005). Antimicrobial activity of flavonoids. Int. J. Antimicrob. Agents 26, 343–356. doi: 10.1016/j.ijantimicag.2005.09.002, PMID: 16323269 PMC7127073

[ref27] Daglia M. Di Lorenzo A. Nabavi S. F. Talas Z. S. Seyed M. (2014). Nabavi polyphenols: well beyond the antioxidant capacity: gallic acid and related compounds as neuroprotective agents: you are what you eat. Curr. Pharm. Biotechnol. 15, 362–372. doi: 10.2174/138920101504140825120737, PMID: 24938889

[ref28] Diegoli S. Adriana L. M. Shakiela B. Ian P. J. Jamie R. L. Jon A. P. (2008). Interaction between manufactured gold nanoparticles and naturally occurring organic macromolecules. Sci. Total Environ. 402, 51–61. doi: 10.1016/j.scitotenv.2008.04.023, PMID: 18534664

[ref29] Eltamany E. E. Elhady S. S. Ahmed H. A. Badr J. M. Noor A. O. Ahmed S. A. (2020). Chemical profiling, antioxidant, cytotoxic activities and molecular docking simulation of *Carrichtera annua* D. (Cruciferae). Antioxidants 9:1286. doi: 10.3390/antiox9121286, PMID: 33339242 PMC7766671

[ref30] Eltamany E. E. Goda M. S. Nafie M. S. Abu-Elsaoud A. M. Hareeri R. H. Aldurdunji M. M. (2022). Comparative assessment of the antioxidant and anticancer activities of *Plicosepalus acacia* and *Plicosepalus curviflorus*: metabolomic profiling and in silico studies. Antioxidants 11:1249. doi: 10.3390/antiox11071249, PMID: 35883740 PMC9311546

[ref31] Eltamany E. E. Nafie M. S. Khodeer D. M. El-Tanahy A. H. H. Abdel-Kader M. S. Badr J. M. (2020). *Rubia Tinctorum* root extracts: chemical profile and management of type II diabetes mellitus. RSC Adv. 10:31214. doi: 10.1039/D0RA90088E, PMID: 35532459 PMC9056425

[ref32] Ezealisiji K. M. Noundou X. S. Ukwueze S. E. (2017). Green synthesis and characterization of monodispersed silver nanoparticles using root bark aqueous extract of *Annona muricata* Linn and their antimicrobial activity. Appl. Nanosci. 7, 905–911. doi: 10.1007/s13204-017-0632-5

[ref33] Fatima N. Hafizur R. M. Hameed A. Ahmed S. Nisar M. Kabir N. (2017). Ellagic acid in *Emblica officinalis* exerts antidiabetic activity through the action on β-cells of pancreas. Eur. J. Nutr. 56, 591–601. doi: 10.1007/s00394-015-1103-y, PMID: 26593435

[ref34] Ferreira I. C. F. R. Baptista P. Vilas-Boas M. Barros L. (2007). Free-radical scavenging capacity and reducing power of wild edible mushrooms from Northeast Portugal: individual cap and stipe activity. Food Chem. 100, 1511–1516. doi: 10.1016/j.foodchem.2005.11.043

[ref35] Fossati P. Prencipe L. (1982). Serum triglycerides determined colorimetrically with an enzyme that produces hydrogen peroxide. Clin. Chem. 28, 2077–2080. doi: 10.1093/clinchem/28.10.2077, PMID: 6812986

[ref36] Gonelimali F. D. Lin J. Miao W. Xuan J. Charles F. Chen M. (2018). Antimicrobial properties and mechanism of action of some plant extracts against food pathogens and spoilage microorganisms. Front. Microbiol. 9:1639. doi: 10.3389/fmicb.2018.01639 30087662 PMC6066648

[ref37] Hanafy A. Badr J. M. (2014). Anti-hyperglycaemic effect of *Phragmanthera austroarabica* A. G. Mill and J. A. Nyberg extract in streptozotocin induced diabetes in rats. Nat. Prod. Res. 28, 2351–2354. doi: 10.1080/14786419.2014.939588 25054215

[ref38] Haque M. Sartelli M. McKimm J. Bakar M. A. (2018). Health care-associated infections–an overview. Infect. Drug. Resist. 11:2321. doi: 10.2147/IDR.S177247 30532565 PMC6245375

[ref39] Ibrahim M. A. Koorbanally N. A. Islam M. S. (2014). Antioxidative activity and inhibition of key enzymes linked to type-2 diabetes (α-glucosidase and α-amylase) by *Khaya Senegalensis*. Acta Pharma. 64, 311–324. doi: 10.2478/acph-2014-0025, PMID: 25296677

[ref40] Jaisinghani R. N. (2017). Antibacterial properties of quercetin. Microbiol. Res. 8:6877. doi: 10.4081/mr.2017.6877

[ref41] Kang J. Liu L. Liu M. Wu X. Li J. J. F. C. (2018). Antibacterial activity of gallic acid against *Shigella flexneri* and its effect on biofilm formation by repressing mdoH gene expression. Food Control 94, 147–154. doi: 10.1016/j.foodcont.2018.07.011

[ref42] Kannan B. Qing W. Wang Y. Liu X. Palvannan T. Wang Y. (2016). Antidiabetic activity of silver nanoparticles from green synthesis using Lonicera japonica leaf extract. RSC Adv. 6, 40162–40168. doi: 10.1039/C5RA24391B

[ref43] Khan H. A. Ahmad A. Mehboob R. J. A. P. J. O. T. B. (2015). Nosocomial infections and their control strategies. Asian Pac. J. Trop. Biomed. 5, 509–514. doi: 10.1016/j.apjtb.2015.05.001

[ref44] Khedr A. I. M. Farrag A. F. S. Nasr A. M. Swidan S. A. Nafie M. S. Abdel-Kader M. S. (2022). Abdelhameed. R.F.a. comparative estimation of the cytotoxic activity of different parts of *Cynara scolymus L.*: crude extracts versus green synthesized silver nanoparticles with apoptotic investigation. Pharmaceutics 14:2185. doi: 10.3390/pharmaceutics14102185, PMID: 36297619 PMC9610270

[ref45] Khedr A. I. M. Goda M. S. Farrag A. F. S. Nasr A. M. Swidan S. A. Nafie M. S. (2022). Silver nanoparticles formulation of flower Head’s polyphenols of *Cynara scolymus* L.: a promising candidate against prostate (PC-3) cancer cell line through apoptosis activation. Molecules 27:6304. doi: 10.3390/molecules27196304, PMID: 36234842 PMC9572662

[ref46] Khodeer D. M. Bilasy S. E. Farag N. E. Mehana A. E. Elbaz A. A. (2019). Sitagliptin protects diabetic rats with acute myocardial infarction through induction of angiogenesis: role of IGF-1 and VEGF. Can. J. Physiol. Pharmacol. 97, 1053–1063. doi: 10.1139/cjpp-2018-0670, PMID: 31116952

[ref47] Khodeer D. M. Zaitone S. A. Farag N. E. Moustafa Y. M. (2016). Cardioprotective effect of pioglitazone in diabetic and non-diabetic rats subjected to acute myocardial infarction involves suppression of AGE-RAGE axis and inhibition of apoptosis. Can. J. Physiol. Pharmacol. 94, 463–476. doi: 10.1139/cjpp-2015-0135, PMID: 27119311

[ref48] Kim Y. K. Kim Y. S. Choi S. U. Ryu S. Y. (2004). Isolation of flavonol rhamnosides from *Loranthus tanakae* and cytotoxic effect of them on human tumor cell lines. Arch. Pharm. Res. 27, 44–47. doi: 10.1007/BF02980044, PMID: 14969337

[ref49] Krzyściak W. Jurczak A. Kościelniak D. Bystrowska B. Skalniak A. J. (2014). The virulence of *Streptococcus mutans* and the ability to form biofilms. Eur. J. Clin. Microbiol. Infect. Dis. 33, 499–515. doi: 10.1007/s10096-013-1993-7 24154653 PMC3953549

[ref50] Kucharska J. Ulicna O. Gvozdjkova A. Sumbalova Z. Vancova O. Bozek P. (2004). Regeneration of coenzyme Q9 redox state and inhibition of oxidative stress by rooibos tea (*Aspalathus linearis*) administration in carbon tetrachloride liver damage. Physiol. Res. 53, 515–521. PMID: 15479130

[ref51] Li D. Liu Z. Yuan Y. Liu Y. Niu F. (2015). Green synthesis of gallic acid-coated silver nanoparticles with high antimicrobial activity and low cytotoxicity to normal cells. Process Biochem. 50, 357–366. doi: 10.1016/j.procbio.2015.01.002

[ref52] Liu X. Fang X. Wang S. Wu D. Gao T. Lee Y. W. (2021). The antioxidant methyl gallate inhibits fungal growth and deoxynivalenol production in *Fusarium graminearum*. Food Produc. Process. Nutr. 3:27. doi: 10.1186/s43014-021-00070-0

[ref53] Magaldi S. Mata-Essayag S. De Capriles C. H. Pérez C. Colella M. Olaizola C. (2004). Well diffusion for antifungal susceptibility testing. Int. J. Infect. Dis. 8, 39–45. doi: 10.1016/j.ijid.2003.03.002 14690779

[ref54] Marrez D. A. Abdelhamid A. E. Darwesh O. M. J. F. P. Life S. (2019). Eco-friendly cellulose acetate green synthesized silver nano-composite as antibacterial packaging system for food safety. Food Packag. Shelf Life 20:100302. doi: 10.1016/j.fpsl.2019.100302

[ref55] Matthews D. R. Hosker J. P. Rudenski A. S. Naylor B. A. Treacher D. F. Turner R. C. (1985). Homeostasis model assessment: insulin resistance and beta-cell function from fasting plasma glucose and insulin concentrations in man. Diabetologia 28, 412–419. doi: 10.1007/BF00280883, PMID: 3899825

[ref56] Mortazavi-Derazkola N. Yousefinia S. A. Naghizadeh A. Lashkari S. Hosseinzadeh M. (2021). Green synthesis and characterization of silver nanoparticles using *Elaeagnus angustifolia* bark extract and study of its antibacterial effect. J. Polym. Environ. 29, 3539–3547. doi: 10.1007/s10924-021-02122-5

[ref57] Mrabti H. N. Jaradat N. Fichtali I. Ouedrhiri W. Jodeh S. Ayesh S. (2018). Separation, identification, and antidiabetic activity of catechin isolated from *Arbutus unedo* L. plant roots. Plants 7:31. doi: 10.3390/plants7020031, PMID: 29649130 PMC6027464

[ref58] Muhammad Z. Azeem M. Mumtaz R. Younas M. Adrees M. Zubair E. (2022). Green synthesis and characterization of silver nanoparticles from *Acacia nilotica* and their anticancer, antidiabetic and antioxidant efficacy. Environ. Pollut. 304:119249. doi: 10.1016/j.envpol.2022.119249 35390420

[ref59] Muhammad R. Suleman A. Ahmad P. Khandaker M. U. Alqahtani A. Bradley D. A. (2022). Biogenic synthesis of AgNPs using aqueous bark extract of *Aesculus indica* for antioxidant and antimicrobial applications. Crystals 12:252. doi: 10.3390/cryst12020252

[ref60] Nemčeková K. Veronika S. Jozef S. Pavol G. Ján L. (2022). Gallic acid-coated silver nanoparticles as perspective drug nanocarriers: bioanalytical study. Anal. Bioanal. Chem. 414, 5493–5505. doi: 10.1007/s00216-022-03955-2 35294597 PMC8923963

[ref61] Nguyen T. L. A. Bhattacharya D. J. M. (2022). Antimicrobial activity of Quercetin: An approach to its mechanistic principle. Molecules 27:2494. doi: 10.3390/molecules27082494 35458691 PMC9029217

[ref62] Oliver S. Harsha W. Yuanli L. Shuang Y. Cyrille B. (2018). Enhancing the antimicrobial and antibiofilm effectiveness of silver nanoparticles prepared by green synthesis. J. Mater. Chem. B. 6, 4124–4138. doi: 10.1039/C8TB00907D, PMID: 32255155

[ref63] Oloruntoyin A. Y. Bakare A. A. Badeggi U. M. Jimoh A. A. Lawal A. Mordi M. N. (2022). Recent advances on therapeutic potentials of gold and silver nanobiomaterials for human viral diseases. Curr. Opin. Chem. Biol. 2:100021. doi: 10.1016/j.crchbi.2022.100021 PMC8806017 35815068

[ref64] Omid A. Jafarizadeh-Malmiri H. Jodeiri N. (2018). Eco-friendly microwave-enhanced green synthesis of silver nanoparticles using *Aloe vera* leaf extract and their physico-chemical and antibacterial studies. Green Process. Synth. 7, 231–240. doi: 10.1515/gps-2017-0039

[ref65] Osadebe P. O. Omeje E. O. Nworu S. C. Esimone C. O. Uzor P. F. David E. K. (2010). Antidiabetic principles of *Loranthus micranthus* Linn. Parasitic on *Persea americana*. Asian Pac. J. Trop. Med. 3, 619–623. doi: 10.1016/S1995-7645(10)60150-2

[ref66] Osonga F. J. Akgul A. Miller R. M. Eshun G. B. Yazgan I. Akgul A. (2019). Antimicrobial activity of a new class of phosphorylated and modified flavonoids. ACS Omega 4, 12865–12871. doi: 10.1021/acsomega.9b00077 31460413 PMC6681995

[ref67] Oyaizu M. (1986). Studies on products of browning reaction: Antioxidative activities of products of browning reaction prepared from glucosamine. Jpn. J. Nutr.Diet 44, 307–315. doi: 10.5264/eiyogakuzashi.44.307

[ref68] Pan M. Song Y.-L. Xu J.-M. Gan H.-Z. (2006). Melatonin ameliorates nonalcoholic fatty liver induced by high-fat diet in rats. J. Pineal Res. 41, 79–84. doi: 10.1111/j.1600-079X.2006.00346.x, PMID: 16842545

[ref69] Park J. Cha S. H. Cho S. Park Y. (2016). Green synthesis of gold and silver nanoparticles using gallic acid: catalytic activity and conversion yield toward the 4-nitrophenol reduction reaction. J. Nanopart. Res. 18, 1–13. doi: 10.1007/s11051-016-3466-2

[ref70] Parmar N. Singla N. Amin S. Kohli K. (2011). Study of cosurfactant effect on nanoemulsifying area and development of lercanidipine loaded (SNEDDS) self-nanoemulsifying drug delivery system. Colloids Surf. B Biointerfaces 86, 327–338. doi: 10.1016/j.colsurfb.2011.04.016, PMID: 21550214

[ref71] Patel S. S. Goyal R. K. (2011). Cardioprotective effects of gallic acid in diabetes-induced myocardial dysfunction in rats. Pharm. Res. 3, 239–245. doi: 10.4103/0974-8490.89743, PMID: 22224046 PMC3249782

[ref72] Patel D. K. Kumar R. Laloo D. Hemalatha S. (2012). Diabetes mellitus: an overview on its pharmacological aspects and reported medicinal plants having antidiabetic activity. Asian Pac. J. Trop. Biomed. 2, 411–420. doi: 10.1016/S2221-1691(12)60067-7, PMID: 23569941 PMC3609313

[ref73] Peter E. S. Obi U. N. (2010). Antilipidaemic studies of mistletoe (*Loranthus micranthus*) leaf extracts on diabetic rats. Int. J. Curr. Res. 8, 48–55.

[ref74] Pietta P. G. (2000). Flavonoids as antioxidants. J. Nat. Prod. 63, 1035–1042. doi: 10.1021/np9904509 10924197

[ref75] Prieto P. Pineda M. Aguilar M. (1999). Spectrophotometric quantitation of antioxidant capacity through the formation of a phosphomolybdenum complex: specific application to the determination of vitamin E. Anal. Biochem. 269, 337–341. doi: 10.1006/abio.1999.4019, PMID: 10222007

[ref76] Qanash H. Yahya R. Bakri M. M. Bazaid A. S. Qanash S. Shater A. F. (2022). Anticancer, antioxidant, antiviral and antimicrobial activities of Kei apple (*Dovyalis cafra*) fruit. Sci. Rep. 12:5914. doi: 10.1038/s41598-022-09993-1, PMID: 35396383 PMC8990652

[ref77] Rahman N. Jeon M. Kim Y. S. (2016). Methyl gallate, a potent antioxidant inhibits mouse and human adipocyte differentiation and oxidative stress in adipocytes through impairment of mitotic clonal expansion. Biofactors 42, 716–726. doi: 10.1002/biof.1310, PMID: 27412172

[ref78] Raymond L. L. Sun W. Y. Chen R. Hui C. K. Ho C. M. Luk J. M. (2008). Silver nanoparticles inhibit hepatitis B virus replication. Antivir. Ther. 13, 253–262. PMID: . 18505176

[ref79] Rojas J. J. Ochoa V. J. Ocampo S. A. Muñoz J. F. (2006). Screening for antimicrobial activity of ten medicinal plants used in Colombian folkloric medicine: A possible alternative in the treatment of non-nosocomial infections. BMC Complement Altern. Med. 6, 1–6. doi: 10.1186/1472-6882-6-2 16483385 PMC1395329

[ref80] Rupasinghe H. P. V. Balasuriya N. Wang Y. (2017). “Prevention of type 2 diabetes by polyphenols of fruits” in Nutritional Antioxidant Therapies: Treatments and Perspectives. eds. Kaïs H. Al-Gubory K. H. Laher I. (Berlin: Springer), 447–466.

[ref81] Samarghandian S. Azimi-Nezhad M. Farkhondeh T. (2017). Catechin treatment ameliorates diabetes and its complications in streptozotocin-induced diabetic rats. Dose Resp. 15:1559325817691158. doi: 10.1177/1559325817691158 PMC5305023 28228702

[ref82] Savu O. Ionescu-Tirgoviste C. Atanasiu V. Gaman L. Papacocea R. Stoian I. (2012). Increase in total antioxidant capacity of plasma despite high levels of oxidative stress in uncomplicated type 2 diabetes mellitus. J. Int. Med. Res. 40, 709–716. doi: 10.1177/147323001204000235, PMID: 22613434

[ref83] Sekhon-Loodu S. Rupasinghe H. P. V. (2019). Evaluation of antioxidant, antidiabetic and antiobesity potential of selected traditional medicinal plants. Front. Nutr. 6:53. doi: 10.3389/fnut.2019.00053, PMID: 31106207 PMC6494929

[ref84] Sheela A. Sarada N. C. Vijayaraghavan R. (2013). A possible correlation between antioxidant and antidiabetic potentials of oxovanadium (IV) complexes. Med. Chem. Res. 22, 2929–2937. doi: 10.1007/s00044-012-0287-4

[ref85] Simoes M. Bennett R. N. Rosa E. A. J. N. P. R. (2009). Understanding antimicrobial activities of phytochemicals against multidrug resistant bacteria and biofilms. Nat. Prod. Rep. 26, 746–757. doi: 10.1039/b821648g 19471683

[ref86] Smanthong N. Tavichakorntrakool R. Tippayawat P. Lulitanond A. Pinlaor P. Daduang J. (2022). Anti-Proteus activity, anti-struvite crystal, and phytochemical analysis of *Sida acuta* Burm. F. ethanolic leaf extract. Molecules 27:1092. doi: 10.3390/molecules27031092 35164357 PMC8838957

[ref87] Son S. M. (2012). Reactive oxygen and nitrogen species in pathogenesis of vascular complications of diabetes. J. Diabetes Metabol. 36, 190–198. doi: 10.4093/dmj.2012.36.3.190, PMID: 22737658 PMC3380122

[ref88] Srinivasan K. Viswanad B. Asrat L. Kaul C. L. Ramarao P. (2005). Combination of high-fat diet-fed and low-dose streptozotocin-treated rat: a model for type 2 diabetes and pharmacological screening. Pharmacol. Res. 52, 313–320. doi: 10.1016/j.phrs.2005.05.004, PMID: 15979893

[ref89] Striegel L. Kang B. Pilkenton S. J. Rychlik M. Apostolidis E. (2015). Effect of black tea and black tea pomace polyphenols on α-glucosidase and α-amylase inhibition, relevant to type 2 diabetes prevention. Front. Nutr. 2:3. doi: 10.3389/fnut.2015.00003 25988132 PMC4428358

[ref90] Suriyakalaa U. Antony J. J. Suganya S. Siva D. Sukirtha R. Kamalakkannan S. (2013). Hepatocurative activity of biosynthesized silver nanoparticles fabricated using *Andrographis paniculata*. Colloids Surf. B Biointerfaces 102, 189–194. doi: 10.1016/j.colsurfb.2012.06.039, PMID: 23018020

[ref91] Synytsya A. Monkai J. Bleha R. Macurkova A. Ruml T. Ahn J. (2017). Antimicrobial activity of crude extracts prepared from fungal mycelia. Asian Pac. J. Trop. Biomed. 7, 257–261. doi: 10.1016/j.apjtb.2016.12.011

[ref92] Tasca F. Riccarda A. (2020). Biocide activity of green quercetin-mediated synthesized silver nanoparticles. Nano 10:909. doi: 10.3390/nano10050909, PMID: 32397267 PMC7279244

[ref93] Tatipamula V. B. Kukavica B. (2021). Phenolic compounds as antidiabetic, anti-inflammatory, and anticancer agents and improvement of their bioavailability by liposomes. Cell Biochem. Funct. 39, 926–944. doi: 10.1002/cbf.3667, PMID: 34498277

[ref94] Tomás M. S. J. Ocaña V. S. Wiese B. Nader-Macías M. E. J. J. O. M. M. (2003). Growth and lactic acid production by vaginal *Lactobacillus acidophilus* CRL 1259, and inhibition of uropathogenic *Escherichia coli*. J. Med. Microbiol. 52, 1117–1124. doi: 10.1099/jmm.0.05155-0 14614071

[ref95] Tripathi R. M. Kumar N. Shrivastav A. Singh P. Shrivastav B. R. (2013). Catalytic activity of biogenic silver nanoparticles synthesized by *Ficus panda* leaf extract. J. Mol. Catal. B Enzym. 96, 75–80. doi: 10.1016/j.molcatb.2013.06.018

[ref96] Tu Y. Deng S. Wang Y. Lin X. Yang Z. J. (2022). Adhesive ability of different oral pathogens to various dental materials: An in vitro study. Can. J. Infect. Dis. Med. Microbiol. 2021:9595067. doi: 10.1155/2022/9595067 PMC9359852 35959001

[ref97] Valgas C. Souza S. M. Smânia E. F. Smânia J. A. (2007). Screening methods to determine antibacterial activity of natural products. Brazilian J. Microbiol. 38, 369–380. doi: 10.1590/S1517-83822007000200034

[ref98] Variya B. C. Anita K. B. Snehal S. P. (2020). Antidiabetic potential of gallic acid from *Emblica officinalis*: improved glucose transporters and insulin sensitivity through PPAR-γ and Akt signalling. Phytomedicine 73:152906. doi: 10.1016/j.phymed.2019.152906, PMID: 31064680

[ref99] Waly N. M. Ali A. E. Jrais R. N. (2012). Botanical and biological studies of six parasitic species of family Loranthaceae growing in Kingdom of Saudi Arabia. Int. J. Environ. Sci. 1, 196–205.

[ref100] Xu H. X. Lee S. F. (2001). Activity of plant flavonoids against antibiotic-resistant bacteria. Phytother. Res. 15, 39–43. doi: 10.1002/1099-1573(200102)15:1<39::AID-PTR684>3.0.CO;2-R 11180521

[ref101] Xu Y. Tang G. Zhang C. Wang N. Feng Y. (2021). Gallic acid and diabetes mellitus: its association with oxidative stress. Molecules 26:7115. doi: 10.3390/molecules26237115, PMID: 34885698 PMC8658971

[ref102] Yang D. Wang T. Long M. Li P. (2020). Quercetin: its main pharmacological activity and potential application in clinical medicine. Oxid. Med. Cell Longev. 2020:8825387. doi: 10.1155/2020/8825387 33488935 PMC7790550

[ref103] Yen G. C. Duh P. D. (1994). Scavenging effect of methanolic extracts of peanut hulls on free radical and active oxygen species. J. Agric. Food Chem. 42, 629–632. doi: 10.1021/jf00039a005

[ref104] Yi S. Yi L. Li L. Jin F. Beiyan L. Xuedong Z. (2011). Antibacterial activity of quercetin on oral infectious pathogens. African J. Microbiol. Res. 5, 5358–5361. doi: 10.5897/AJMR11.849

[ref105] Zanwar A. A. Badole S. L. Shende P. S. Hegde M. V. Bodhankar S. L. (2014). Chapter 21 - antioxidant role of catechin in health and disease. Polyphenols Hum. Health Dis. 1, 267–271. doi: 10.1016/C2011-1-09286-X

[ref106] Zeinab S. A. Khodeer D. M. Zaitone S. A. Ahmed A. A. M. Moustafa Y. M. (2020). Exenatide ameliorates experimental non-alcoholic fatty liver in rats via suppression of toll-like receptor 4/NFκB signaling: comparison to metformin. Life Sci. 253:117725. doi: 10.1016/j.lfs.2020.117725 32348835

